# Taxonomic revision of Madagascan species of the *Pheidole fervens* species-group (Hymenoptera, Formicidae)

**DOI:** 10.1371/journal.pone.0244195

**Published:** 2021-01-06

**Authors:** Sebastian Salata, Brian L. Fisher

**Affiliations:** Department of Entomology, California Academy of Sciences, San Francisco, California, United States of America; Nanjing Agricultural University, CHINA

## Abstract

Madagascar, one of the top megadiversity regions, hosts one of the highest numbers of endemic and threatened organisms on earth. One of the most spectacular examples of ant radiation on the island has occurred in the hyperdiverse genus *Pheidole*. To this date, there are 117 described Madagascan *Pheidole* divided into 16 species-groups, and 97% of them are endemic to the island. Only two of these species-groups contain widely distributed invasive species in addition to native, endemic taxa: *megacephala*, and *fervens* species-groups. Here we revise the *fervens* species-group and discuss updated distribution records of its introduced members on Madagascar. We recognize six species belonging to this group, including five new to science: *Pheidole ampangabe* sp. nov., *P*. *arivo* sp. nov., *P*. *comosa* sp. nov., *P*. *indica* Mayr, *P*. *mamirapiratra* sp. nov., and *P*. *mena* sp. nov. Detailed descriptions are supplemented with measurements, diagnoses, identification key, high-resolution images for major and minor worker, and comments on distribution and biology.

## Introduction

The ant genus *Pheidole* is the most speciose within the Formicidae family. It comprises 1151 described species known from all biogeographic regions but Antarctic [1]. Studies on its diversity in the Malagasy region had been neglected for decades until a taxonomic revision of species known from Comoros, Juan de Nova Island, Mauritius, Mayotte, Réunion, and Seychelles [2], followed later by papers focused exclusively on the Madagascan fauna [3–5]. Recent data confirms estimates stated by Fisher & Peeters [6] and reveals exceptional, on the global scale, level of endemism of Malagasy *Pheidole*. So far, there are 130 *Pheidole* species from this region, and as much as 97% of them are considered endemic. The number, however, is still not complete, and we estimate that there are approximately 20 taxa awaiting descriptions in the forthcoming revisions.

The Malagasy species of *Pheidole* have conspicuously dimorphic worker caste and are characterized by the combination of the following characters [7]: 12-segmented antennae with a strongly defined, 3 segmented club; major worker with disproportionately enlarged head and at least one set of hypostomal teeth on posterior margin of buccal cavity; large clypeus broadly inserted posteriorly between the frontal lobes; triangular mandible, large and essentially edentate apart from the near basal tooth and the apical and sub-apical teeth in majors, more delicate and serially dentate in minors; palp formula 3,2, or 2,2; frontal carinae short in all minors and most majors, in majors of some species the carinae extend almost to the posterior margin of the head; antennal scrobe absent in minors but variably developed in majors; in majors of some species distinct scrobe are present that extend above the eyes; eyes present, located at most often at the midlength of the head capsule in minors, in majors of some species located below the midlength of the head capsule; pronotum and anterior mesonotum swollen and convex in profile, usually dome-like; in profile the propodeal dorsum on a much lower level than the top of the convex promesonotum; promesonotal suture represented across the dorsum in some species by a weak impression, or by a narrow line, in others vestigial or entirely absent; propodeum unarmed to bispinose; propodeal spiracle large, its orifice circular or nearly so, located at or slightly behind the midlength of the sclerite; abdominal segment 2 (petiole) with a distinct anterior peduncle; abdominal tergite 4 (first gastral) broadly overlaps the sternite on the ventral gaster; main pilosity of dorsal head and body simple, may be sparse.

Taxonomic knowledge on Madagascan *Pheidole* has greatly improved in recent times, and there are only three species-groups pending revisions: *lucida*, *megacephala*, and *fervens*. While the *lucida* species-group appears to consist of only native species, the remaining two groups comprise a combination of native, endemic taxa and widely distributed invasive species. The *megacephala* species-group is most likely of Afrotropical origin but displays a high diversity in the Malagasy [8]. Its representative: *Pheidole megacephala* (Fabricius), is recognized as one of the most destructive, cosmopolitan pest listed among 100 worst invasive species [9]. The *fervens* species-group is native to Indoaustralia and consist of several species [8, 10], of which two established colonies beyond their native range: *Pheidole fervens* Smith and *Pheidole indica* Mayr [8]. Both species probably negatively impact native arthropods [8] and have confirmed presence in the Malagasy [2]. So far, the only verified records of *P*. *fervens* come from Mauritius [2], but we can’t exclude its possible presence in urban and agricultural parts of Madagascar. While *P*. *indica* is a common pest in anthropogenic sites across the whole Malagasy, including Madagascar. Our study revealed that the island also hosts 5 additional, undescribed members of the *fervens* species-group. All the new species have distribution limited to Madagascar and are considered as endemic for this island. Below, we present a taxonomic revision of the *fervens* species-group from Madagascar and discuss general patterns of the *Pheidole* diversity on the island.

## Materials and methods

Ant samples used in this study comply with the regulations for export and exchange of research samples outlined in the Convention of Biology Diversity and the Convention on International Trade in Endangered Species of Wild Fauna and Flora. For fieldwork conducted in Madagascar, permits to research, collect and export ants were obtained from the Ministry of Environment and Forest as part of an ongoing collaboration between the California Academy of Sciences and the Ministry of Environment and Forest, Madagascar National Parks and Parc Botanique et Zoologique de Tsimbazaza. Authorization for export was provided by the Director of Natural Resources. Approval Numbers: No. 0142N/EA03/MG02, No. 340N-EV10/MG04, No. 69 du 07/04/06, No. 065N-EA05/MG11, No. 047N-EA05/MG11, No. 083N-A03/MG05, No. 206 MINENVEF/SG/DGEF/DPB/SCBLF, No. 0324N/EA12/MG03, No. 100 l/fEF/SG/DGEF/DADF/SCBF, No. 0379N/EA11/MG02, No. 200N/EA05/MG02

The present study was conducted on 336 specimens belonging to 175 samples collected in Madagascar and deposited in the California Academy of Sciences, San Francisco, California, U.S.A. All specimen data are listed in S2 Table and additionally are freely accessible on AntWeb (http://www.antweb.org). Each specimen used in this study can be traced by a unique specimen identifier affixed to the pin (e.g. CASENT0236484).

Repositories. Collections are referred to by the following acronyms:

**CASC**–California Academy of Sciences, San Francisco, California, USA;

**MHNG**–Muséum d’Historie Naturelle, Geneva, Switzerland;

**PBZT**–Parc Botanique et Zoologique de Tsimbazaza, Antananarivo, Madagascar.

All observations and measurements were taken using a pin-holding stage, permitting rotations around the X, Y, and Z axes at magnifications from 32× to 100× with a Leica MZ12.5 microscope and an orthogonal crosshair micrometre, at an accuracy of 0.01 mm to approximately 0.005 mm. All measurements are presented in mm units as minimum and maximum values, with the arithmetic mean in parentheses. Photographs were taken using a JVC KY-75 or Leica DFC450 digital camera with a Leica Z16 APO microscope and Leica Application Suite software (v3.8). Unless stated otherwise, photographs were taken by Michele Esposito, and material was collected by B. L. Fisher and his collaborators and is stored in CASC. Images of specimens and data of all pinned specimens examined in the present contribution are available online on AntWeb (www.AntWeb.org) and accessible using the unique CASENT identifying specimen code. Measurements and indices are in line with Salata and Fisher [3–5] and are mostly the same as in Longino [11, 12] and several other revisions [2, 13–15]. The general morphological terminology follows Wilson [16] and Longino [11, 12]. The surface sculpturing glossary follows Harris [17].

Our recognition of species follows the biological species concept and species boundaries are based on comparative morphology and known geographic distributions of investigated taxa. Where sympatric populations exhibit consistently different phenotypes, they are considered different species. Species described based on the single nest sample exhibit distinct and unique set of morphological features allowing their separation from other Madagascan *Pheidole* species.

Pilosity inclination degree follows that used in Wilson [18]. Appressed (0–5°) hairs run parallel or nearly parallel to the body surface. Decumbent hairs stand 10–40°, subdecumbent hair stand ~ 45° from the surface°, suberect hairs bend about 10°–20° from vertical, and erect hairs stand vertical or nearly vertical.

### Measurements and indices

**Fig 1:**
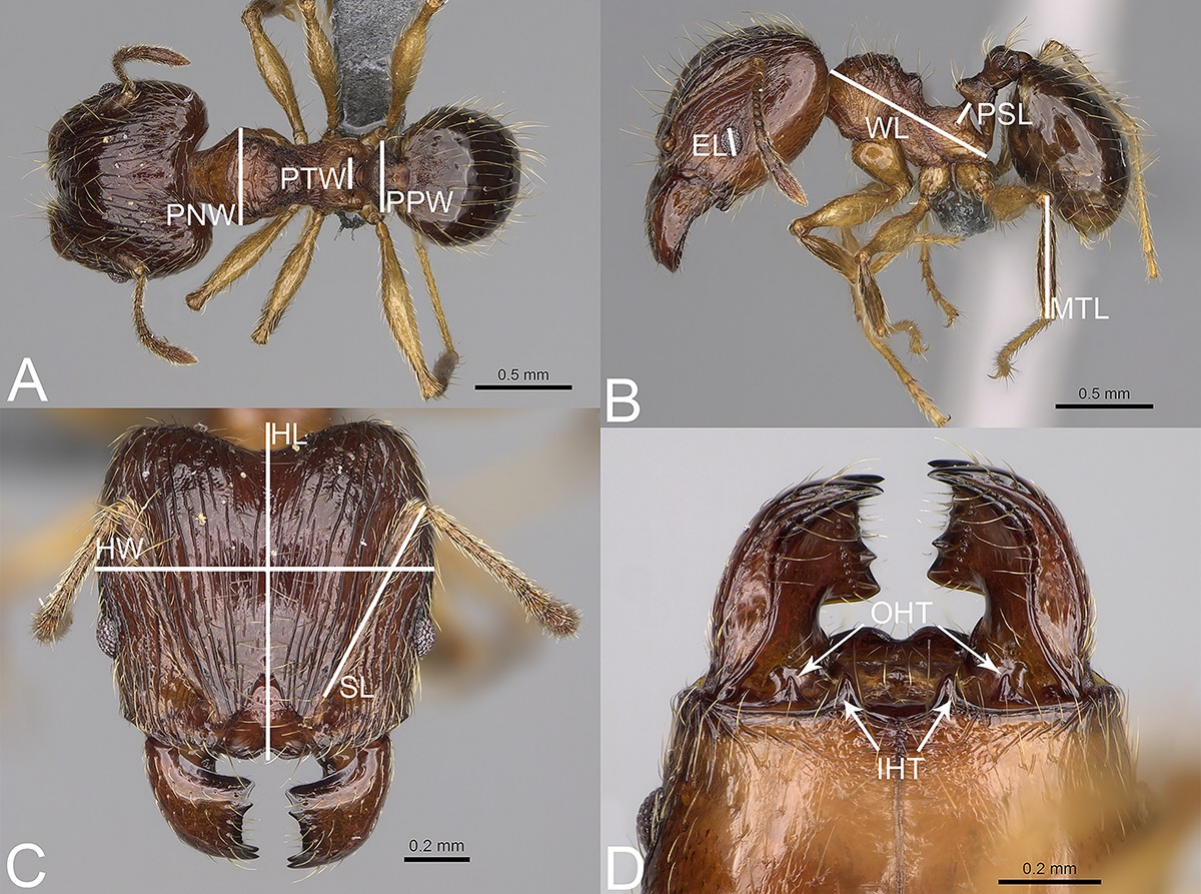
*Pheidole arivo*, illustrations of measurements (A–C). A. Dorsal view. B. Profile. C. Full-face view. D. Inner hypostomal teeth (IHT) and outer hypostomal teeth (OHT).

Measurements (Fig 1)

**EL**–eye length; measured along the maximum vertical diameter of the eye;

**HL**–maximum distance from the midpoint of the anterior clypeal margin to the midpoint of the posterior margin of the head, measured in full-face view; in majors from midpoint of tangent between anteriormost position of clypeus to midpoint of tangent between posteriormost projection of the vertex;

**HW**–head width; measured in full-face view, at widest point of the head, directly above the eyes;

**MTL**–metatibia length; straight line length of the metatibia measured from the constriction immediately before its proximal insertion to its distalmost point, excluding the bristles or spines;

**PNW**–pronotum width; maximum width of promesonotum measured in dorsal view;

**PPW**–postpetiole width; maximum width of postpetiole in dorsal view;

**PSL**–propodeal spine length; measured from the centre of the propodeal spiracle to the tip of the propodeal spine in lateral view;

**PTW**–petiole width; maximum width of petiole in dorsal view;

**SL**–scape length; maximum straight-line length of scape excluding the basal condylar bulb;

**WL**–mesosoma length (Weber’s length); diagonal length of mesosoma in lateral view from the anterior point of the pronotal slope and excluding the neck, to the posteroventral margin of the propodeum.

Indices

**CI**–cephalic index: HW / HL * 100;

**MTI**–tibia index: MTL / HW * 100;

**SI**–scape index: SL / HW * 100;

**PNI**–pronotum index: PNW / HW * 100;

**PPI**–postpetiole width index: PPW / PTW * 100;

**PSLI**–propodeal spine index: PSL / HW * 100.

Abbreviations

**m**.–male; **q**.–gyne; **s**–major worker; **w**.–minor worker.

**Fig 2:**
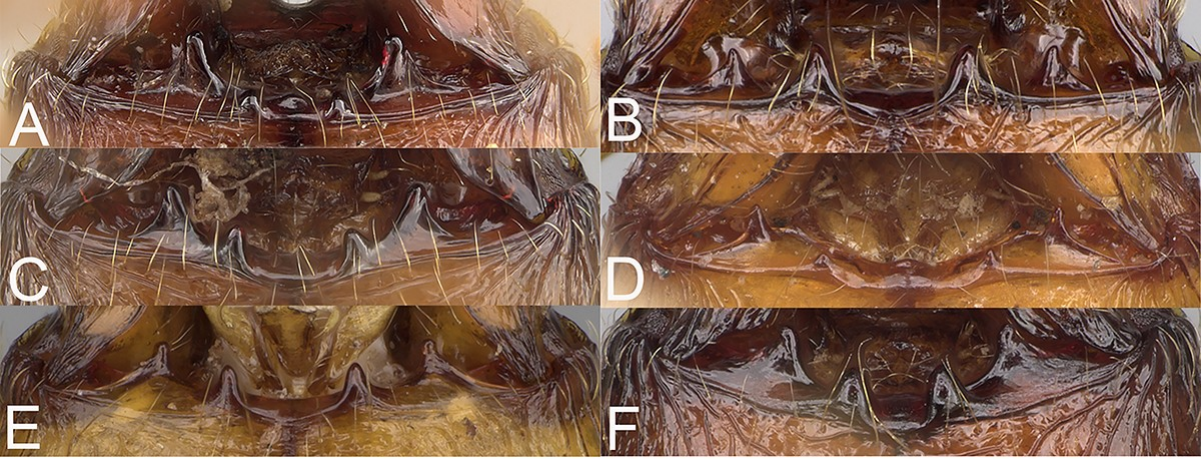
Major worker, hypostomal teeth. *Pheidole ampangabe* (A). *P*. *arivo* (B). *P*. *comosa* (C). *P*. *indica* (D). *P*. *mamirapiratra* (E). *P*. *mena* Head (F).

Distribution maps (Fig 2) were generated using tmap v2.2 package on R v3.5. R Core Team [19].

### Nomenclatural acts

The electronic edition of this article conforms to the requirements of the amended International Code of Zoological Nomenclature, and hence the new names contained herein are available under that Code from the electronic edition of this article. This published work and the nomenclatural acts it contains have been registered in ZooBank, the online registration system for the ICZN. The ZooBank LSIDs (Life Science Identifiers) can be resolved and the associated information viewed through any standard web browser by appending the LSID to the prefix “http://zoobank.org/”. The LSID for this publication is: urn:lsid:zoobank.org:pub: 47A76C39-8950-4785-86E5-EB058E262702. The electronic edition of this work was published in a journal with an ISSN, and has been archived and is available from the following digital repositories: PubMed Central, LOCKSS.

## Results

### Synopsis of Madagascan members of the *Pheidole fervens* species-group

*Pheidole ampangabe* sp. nov.

*Pheidole arivo* sp. nov.

*Pheidole comosa* sp. nov.

*Pheidole indica* Mayr, 1879

*Pheidole mamirapiratra* sp. nov.

*Pheidole mena* sp. nov.

### Species accounts

Repetitive characters occurring in the majority of species have been omitted. Unless stated otherwise, the following descriptions apply to all species treated here:

#### Major worker

Dorsal face of the head in lateral view not depressed posteriorly; frontal lobe absent; head in full-face view with distinct median concavity; antenna 12-segmented, with a 3-segmented club; masticatory margin of mandible with large, stout apical and preapical teeth, followed by a long diastema and then a short and crenulate tooth just before the rounded basal angle; the outer surface of mandible mostly smooth and shining, sometimes with weak and sparse puncta; promesonotum strongly convex, well above the level of propodeum; promesonotum short, angular, and low; posterior mesonotum steep; petiolar peduncle long, with small horizontal lobe on its basal part; petiolar node low, triangular, with rounded and thin top, pilosity sparse and erect; postpetiole short with slightly convex dorsum, pilosity long, sparse, and erect.

#### Minor worker

Antennal socket shallow and surrounded by a few indistinct, thin and curved outward rugae; frontal lobe absent; head in full-face view oval; posterior and anterior of eyes convex; scape, when laid back, exceeding the posterior head margin by two-fifths of its length, pilosity dense, subdecumbent to erect; antenna 12-segmented, with a 3-segmented club; clypeus smooth and shiny, its anterior margin regularly convex; clypeus with median longitudinal carina absent, two lateral longitudinal carinae absent; humeral tubercle not developed into projection; promesonotum well above the level of propodeum; posterior mesonotum steep; petiolar peduncle with ventral face slightly convex, node low, triangular, and small, with few short, erect setae; postpetiole with few short, erect setae; gaster smooth and shiny.

### Revision of the *Pheidole fervens* group from Madagascar

#### Diagnosis. Major worker

**Fig 3:**
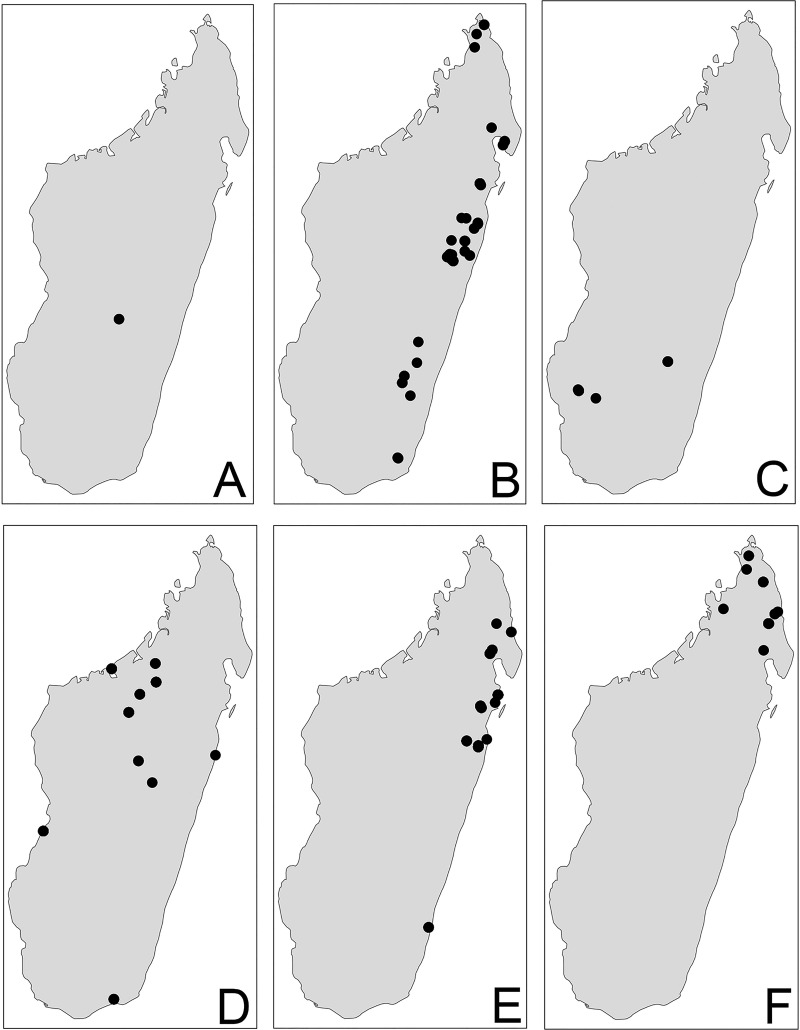
Distribution. *Pheidole ampangabe* (A). *P*. *arivo* (B). *P*. *comosa* (C). *P*. *indica* (D). *P*. *mamirapiratra* (E). *P*. *mena* Head (F).

Postpetiole in profile without conspicuous ventral convexity; antennal socket shallow; frontal lobe absent or indistinct; propodeal spine short, with wide base; head in full-face view sub-rectangular with lateral margins from relatively straight to slightly convex, not or slightly widening posteriorly; head in lateral view sub-oval with distinctly convex margins; occipital lobe most often entirely sculptured (except *P*. *ampangabe*); frons costulate to rugocostulate; antennal scrobe distinct but shallow, always sculptured with at least fine rugae; costulae or rugocostulae on head thick to thin; both inner and outer hypostomal teeth present (Fig 3); promesonotum short, angular and low; postpetiole in dorsal view with lateral margins medially with two dentate projections; gaster at least partially shagreened (except *P*. *mena*, *P*. *indica*, and *P*. *fervens*); mesonotal process most often distinct. **Minor worker.** Postpetiole in profile without conspicuous ventral convexity; antennal socket shallow; frontal lobe absent or indistinct; propodeal spine indistinct to moderate; promesonotum in lateral view never box-like; anterior mesonotum slightly to distinctly concave; posterior mesonotum steep or smoothly declining towards propodeum; posterior region of head never forming neck; promesonotum low and short, arched; head and mesosoma with poorly developed sculpture.

### Key to the Madagascan members of the *P*. *fervens* group

Despite the absence of confirmed records of *P*. *fervens* from Madagascar we decided to include this species in the key. So far, the only verified records from the Malagasy region come from Mauritius [2], but we can’t exclude its possible presence in strongly disturbed by human activity parts of the island.

**Fig 4:**
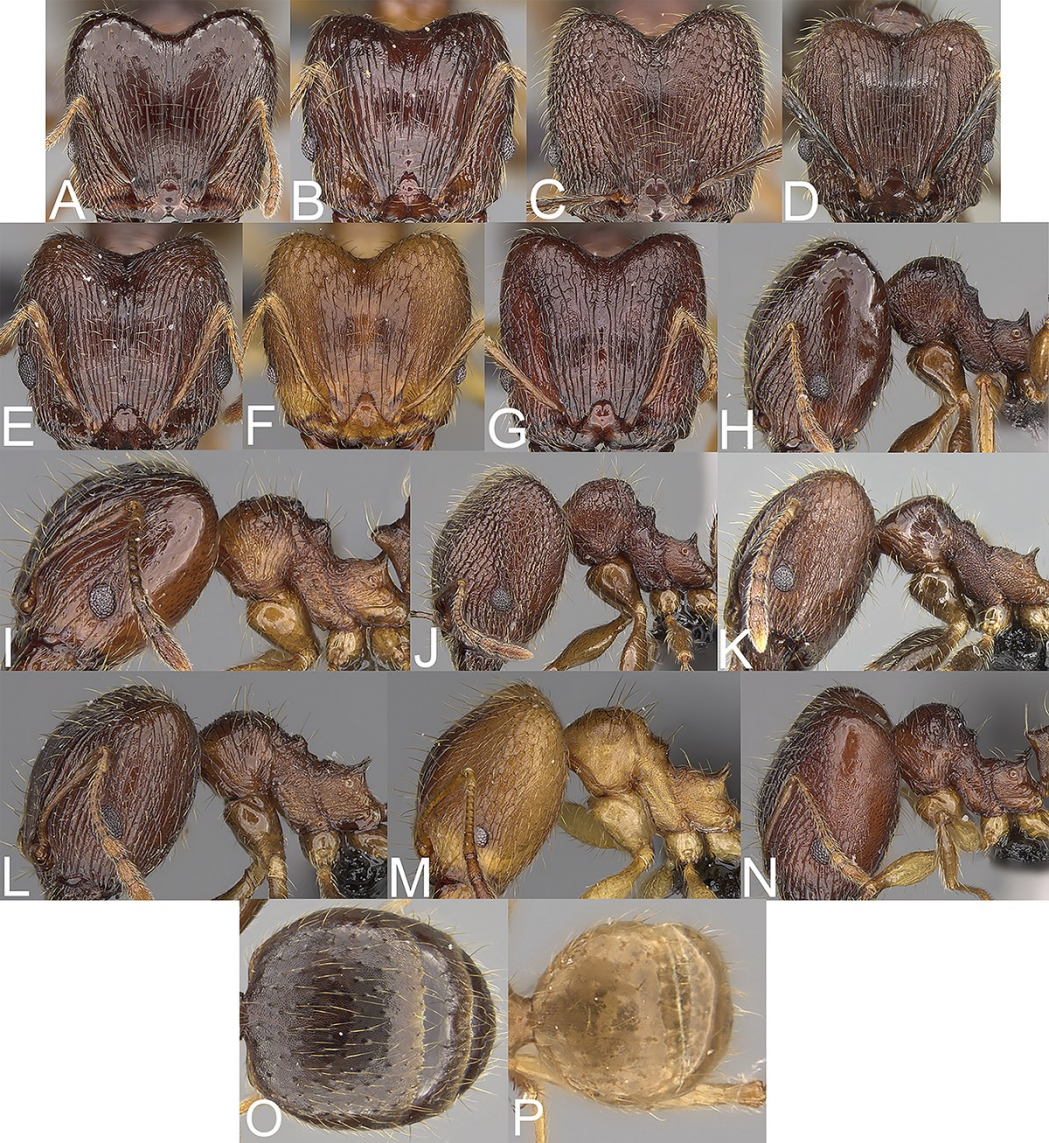
Major worker. *Pheidole ampangabe*, Head (A). Profile (H). Gaster (O). *P*. *arivo*, Head (B). Profile (I). *P*. *comosa*, Head (C). Profile (J). *P*. *fervens*, Head (D). Profile (K). *P*. *indica*, Head (E). Profile (L). *P*. *mamirapiratra* Head (F). Profile (M). Gaster (P). *P*. *mena* Head (G). Profile (N).

**Fig 5:**
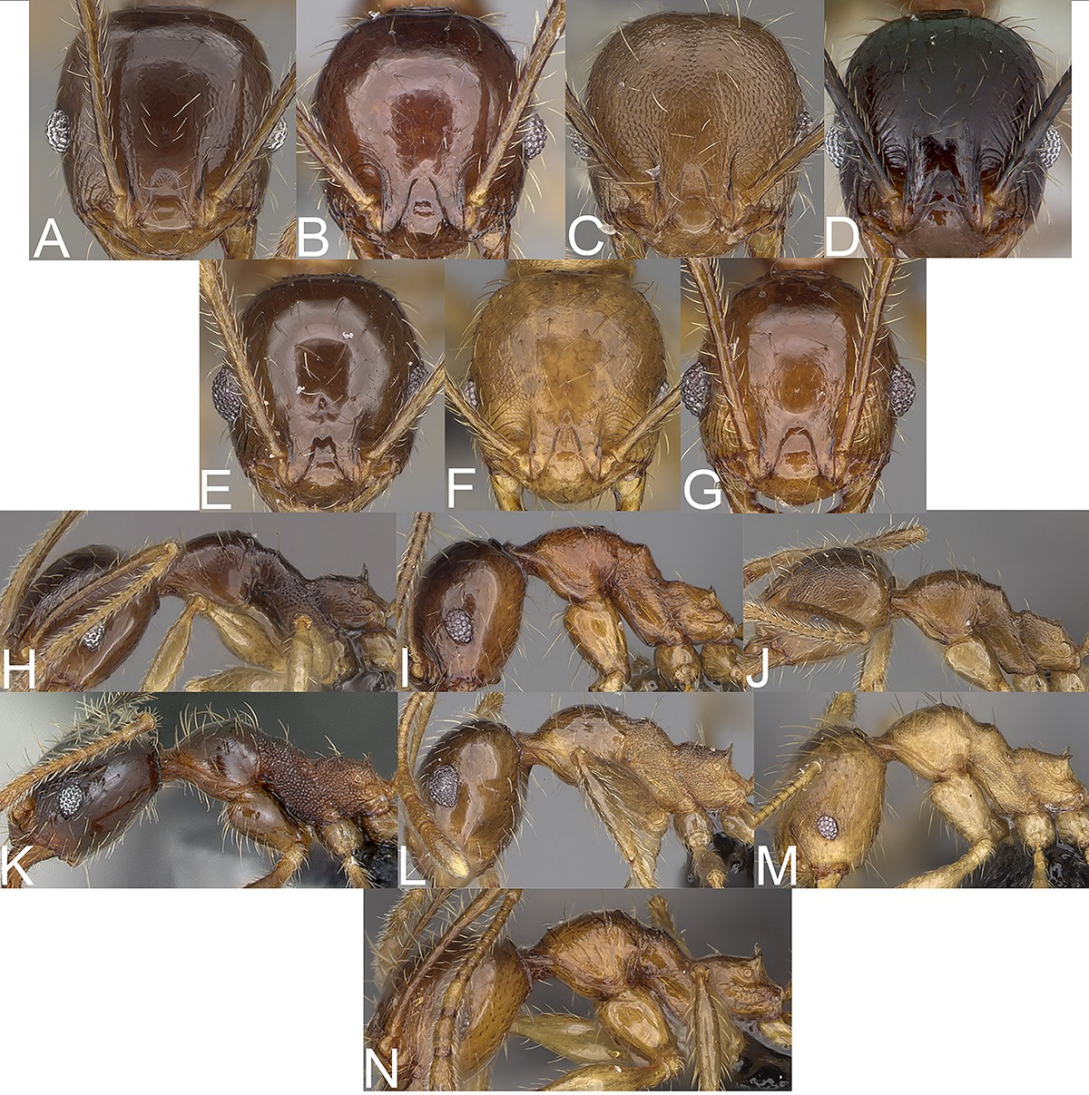
Minor worker. *Pheidole ampangabe*, Head (A). Profile (H). *P*. *arivo*, Head (B). Profile (I). *P*. *comosa*, Head (C). Profile (J). *P*. *fervens*, Head (D). Profile (K). *P*. *indica*, Head (E). Profile (L). *P*. *mamirapiratra* Head (F). Profile (M). *P*. *mena* Head (G). Profile (N).

1. Major worker. Margins of the head with dense, long and suberect to erect pilosity, antennal scrobe costulate, with distinctly rugopunctate interspaces, frons with dense costulae, interspaces between costulae rugulate (Fig 4C and 4J). Minor worker. Head sculpture shiny and sparsely punctate, sides posterolateral from eyes entirely to mostly smooth (Fig 5C and 5J). ……….. ***Pheidole comosa***
  - Major worker. Margins of the head with sparser, shorter and decumbent to erect pilosity, antennal scrobe with different sculpture, frons with sparser costulae, interspaces between costulae smooth to indistinctly rugulate or punctate (Fig 4A and 4B, 4D–4I and 4K–4N). Minor worker. Head smooth or mostly smooth with indistinct puncta (Fig 5A and 5B, 5D–5I and 5K–5N). ……….. 2.
2. Major worker. Occipital lobe smooth, first gastral tergite entirely shagreened (Fig 4A, 4H and 4O). Minor worker. Head with indistinct and sparse puncta, body brown and propodeum with indistinct puncta (Fig 5A and 5H). ……….. ***Pheidole ampangabe***
  - Major worker. Occipital lobe sculptured, first gastral tergite at least partly smooth (Fig 4B, 4D–4G, 4I and 4K–4N). Minor worker. Head smooth, if frons with indistinct and sparse puncta then body yellow to yellowish-brown and propodeum densely and distinctly punctate (Fig 5B, 5D–5G, 5I and 5K–5N). ………….. 3.
3. Major worker. Body reddish-brown, head in lateral view more rectangular, promesonotum high (Fig 4G and 4N). Minor worker. Mesosoma mostly smooth with thick and sparse rugae, anterior promesonotum slightly concave, posterior promesonotum smoothly declining towards propodeum (Fig 5G and 5N). ………….. ***Pheidole mena***
  - Major worker. Body yellow never reddish-brown, head in lateral view distinctly oval, promesonotum lower (Fig 4B, 4D–4F, 4I and 4K–4M). Minor worker. Mesosoma always with distinct puncta at least on propodeum, if puncta are indistinct then posterior promesonotum steep (Fig 5B, 5D–5F, 5I and 5K–5M). ………….. 4.
4. Major worker. Sides posterolateral from eyes smooth, mesonotal process distinct (Fig 4B and 4I). Minor worker. Posterior mesonotum steep, katepisternum entirely or mostly smooth (Fig 5B and 5I). ……………….. ***Pheidole arivo***
  - Major worker. Sides posterolateral from eyes sculptured, mesonotal process less distinct (Fig 4D–4F and 4K–4M). Minor worker. Posterior mesonotum slightly steep to smoothly declining toward propodeum, katepisternum punctate (Fig 5D–5F and 5K–5M). ……. 5.
5. Major worker. Antennal scrobe with dense network of microrugulae and additional sparse, thick rugocostulae, base of first gastral tergite shagreened (Fig 4F, 4M and 4P). Minor worker. Head with indistinct and sparse puncta, sides posterolateral from eyes smooth to indistinctly punctate, promesonotum with indistinct sculpture (Fig 5F and 5M). ……… ***Pheidole mamirapiratra***
  - Major worker. Antennal scrobe costulate with smooth to punctate interspaces, gaster smooth (Fig 4D and 4E, 4K and 4L). Minor worker. Head smooth, promesonotum smooth (Fig 5D and 5E, 5K and 5L). …….. 6.
6. Major worker. Interspaces between costulae on antennal scrobe distinctly punctate, lateral margins of the head with dense and suberect to erect setae (Fig 4D and 4K). Minor worker. Metanotal groove shallow to indistinct, posterior mesonotum usually smoothly declining towards propodeum and with no protuberance (Fig 5D and 5K). …… ***Pheidole fervens***
  - Major worker. Interspaces between costulae on antennal scrobe smooth to indistinctly punctate, lateral margins of the head with sparser and appressed to subdecumbent setae (Fig 4E and 4L). Minor worker. Metanotal groove deeper, posterior mesonotum usually steeper and with protuberance (Fig 5E and 5L). …… ***Pheidole indica***

### *Pheidole ampangabe* sp. nov.

urn:lsid:zoobank.org:act:808B2B86-7122-4818-9B99-6A0F9815EF0E

**Fig 6:**
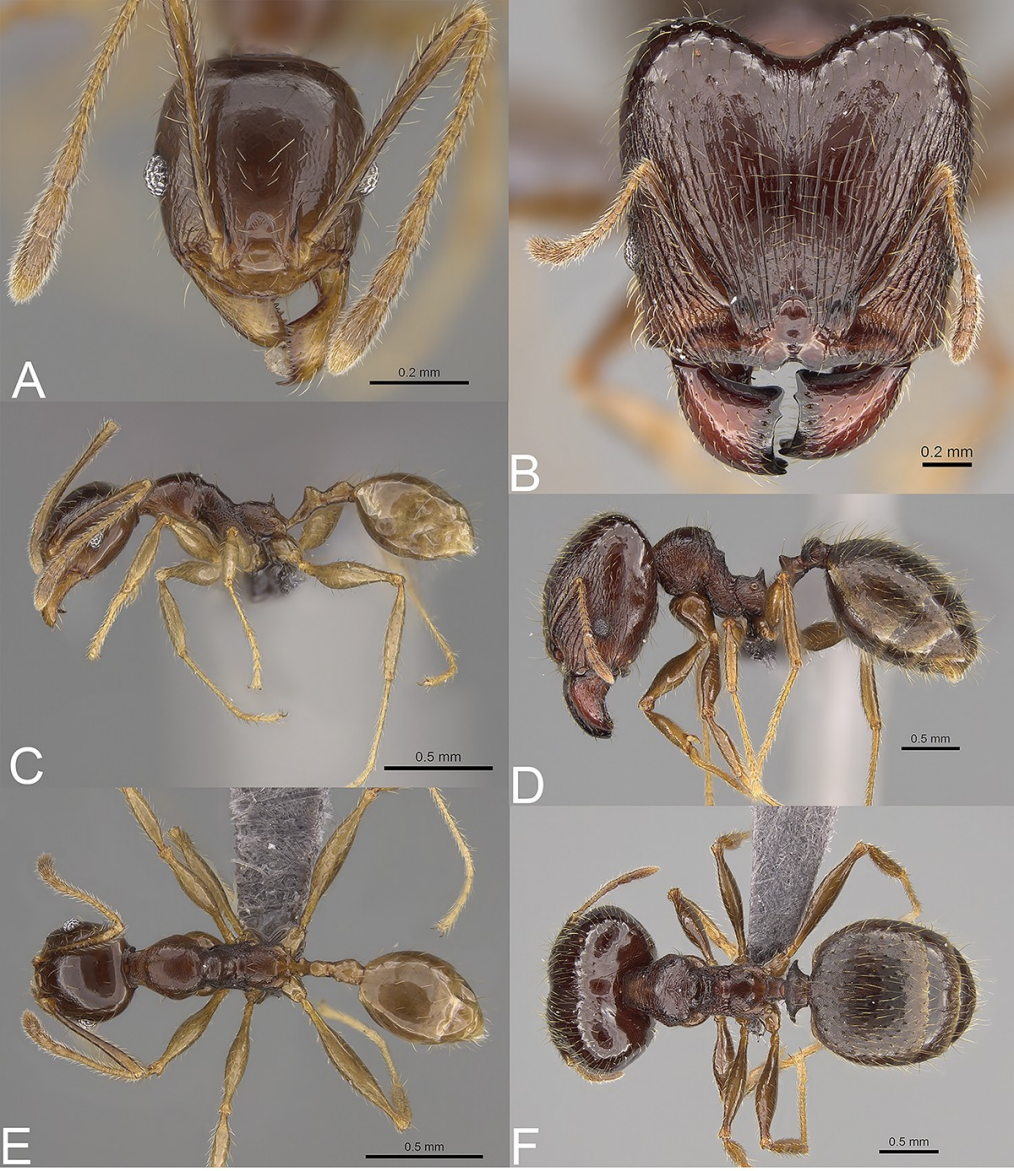
*Pheidole ampangabe*, full-face view (A), profile (C), and dorsal view (E) of paratype minor worker (CASENT0236484) and full-face view (B), profile (D), and dorsal view (F) of holotype major worker (CASENT0923264).

Figs 2A, 3A, 4A, 4H, 5A, 5H and 6A–6F

HOLOTYPE: 1s., Madagascar, Fianarantsoa, Ampangabe IV Non-Protected Area, 21.37 km W Itremo, -20.61278 46.60774, 1417 m, 21-Mar-2010, savannah woodland, under rotten log, A. Ravelomanana leg., ARA0859, CASENT0923264 (CASC). PARATYPES: 1w., the same data as holotype, CASENT0236484 (CASC).

#### Geographic range

Madagascar, Fianarantsoa, Ampangabe IV Non-Protected Area.

#### Diagnosis. Major worker

Head, in full-face view sub-rectangular, slightly widening posteriorly. Margins of the head with dense, short and suberect to erect pilosity. Antennal scrobe densely rugocostulate; interspaces between rugocostulae distinctly punctate. Frons with thick and sparse costulae; interspaces between costulae mostly smooth or indistinctly punctate. Sides posterolateral from eyes distinctly shagreened and smooth apically. Occipital lobe smooth. Inner hypostomal tooth distinct, small, closely spaced, bulge-like, with rounded top; outer hypostomal tooth lobe-like, distinctly bigger and wider than inner hypostomal teeth, top directed upward; median tooth present, indistinct. Mesosoma with dense rugoreticulae; promesonotal dorsum with sparser sculpture and partially smooth. Gaster shagreened. Body brown, legs and scape yellowish brown. **Minor worker.** Head shiny and with indistinct and sparse puncta; sides posterolateral from eyes smooth. Mesosoma shiny; promesonotum and anepisternum with indistinct and sparse puncta; lateral sides of pronotum partially smooth; katepisternum and propodeum with more distinct but still sparse puncta. Head and mesosoma brown; gaster, antennae, and legs yellowish-brown.

#### Description. Major worker

Measurements (n = 1): HL: 1.76; HW: 1.78; SL: 0.91; EL: 0.2; WL: 1.41; PSL: 0.22; MTL: 0.98; PNW: 0.71; PTW: 0.25; PPW: 0.69; CI: 98.9; SI: 50.9; PSLI: 12.3; PPI: 36.0; PNI: 39.6; MTI: 55.2.

#### Head

In full-face view sub-rectangular, slightly widening posteriorly, with anterior and posterior margins slightly convex (Fig 6B). In lateral view sub-oval. Inner hypostomal tooth visible. Margins of the head with dense, short and suberect to erect pilosity; head dorsum with dense, long and decumbent to erect pilosity. Antennal scrobe distinct but shallow; densely rugocostulae; interspaces between rugocostulae distinctly punctate. Frons with thick and sparse costulae; interspaces between costulae mostly smooth indistinctly punctate. Sides posterolateral from eyes distinctly shagreened and smooth apically. Occipital lobe smooth. Gena with sparse and thick costulae; interspaces between costulae distinctly punctate. Centre of clypeus smooth and shiny, lateral margin with indistinct rugulae; median notch present, wide, and deep; median longitudinal carina present; lateral longitudinal carinae absent. Scape, when laid back, exceeding the midlength of the head by two-fifths of its length; pilosity subdecumbent to erect (Fig 6B and 6D). Inner hypostomal tooth distinct, small, closely spaced, bulge-like, with rounded top; outer hypostomal tooth lobe-like, distinctly bigger and wider than inner hypostomal teeth, top directed upward; inner and outer hypostomal teeth closely spaced and not connected by concavity; median tooth present, indistinct (Fig 2A). **Mesosoma.** In lateral view, mesonotal process moderate, tubercle-like; promesonotal groove absent; metanotal groove absent; propodeal spine moderate, with a wide base and acute top; humeral tubercle laterally weakly produced (Fig 6D). Surface shiny; densely rugoreticulae; promesonotal dorsum with sparser sculpture. Pilosity dense, long, and erect (Fig 6D and 6F). **Petiole.** Shiny and shagreened; node, in rear view dorsoventrally concave (Fig 6D and 6F). **Postpetiole.** Shiny and shagreened; dorsum with dense sculpture; in dorsal view oval, lateral margins medially with two distinct, dentate projections (Fig 6D and 6F). **Gaster.** Shiny and shagreened; pilosity dense, long, and erect (Fig 6D and 6F). **Colour.** Brown, legs and scape yellowish-brown (Fig 6D and 6F).

#### Description. Minor worker

Measurements (n = 1): HL: 0.63; HW: 0.54; SL: 0.76; EL: 0.14; WL: 0.83; PSL: 0.1; MTL: 0.61; PNW: 0.37; PTW: 0.1; PPW: 0.14; CI: 116.2; SI: 140.0; PSLI: 15.7; PPI: 68.6; PNI: 69.0; MTI: 112.9.

#### Head

Occipital margin slightly convex; occipital carina absent (Fig 6A). Pilosity sparse, long, and suberect to erect. Sculpture shiny with indistinct and sparse puncta, sides posterolateral from eyes smooth (Fig 6A and 6C). **Mesosoma.** In lateral view, promesonotum low and short, arched; promesonotal groove absent; metanotal groove indistinct; anterior mesonotum concave; posterior mesonotum smoothly declining toward propodeum; propodeal spine moderate and thin (Fig 6C). Sculpture shiny; promesonotum and anepisternum with indistinct and sparse puncta; lateral sides of pronotum partially smooth; katepisternum and propodeum with more distinct but still sparse puncta. Pilosity sparse, long, and erect (Fig 6C and 6E). **Postpetiole.** Convex; in dorsal view slightly widening posteriad (Fig 6C and 6E). **Gaster.** With sparse, erect pilosity (Fig 6C and 6E). **Colour.** Head and mesosoma brown; gaster, antennae, and legs yellowish brown (Fig 6C and 6E).

#### Biology

The species was collected at 1417 m in elevation, in savannah woodland. The nest was located under a rotten log.

#### Comments

*Pheidole ampangabe* is probably endemic to savannah woodlands of Ampangabe and its distribution doesn’t overlap with other members of the group. Majors of *P*. *ampangabe* can be easily separated from other species based on smooth occipital lobe and entirely punctate gaster. Minors, with indistinctly punctate head sculpture, are most similar to *P*. *mamirapiratra* but they differ in brown body coloration and partially smooth promesonotum. In contrast, minors of *P*. *mamirapiratra* have entirely sculptured promesonotum and yellow to yellowish brown body.

**Etymology.** After the locus typicus.

### *Pheidole arivo* sp. nov.

urn:lsid:zoobank.org:act:62B1D5B5-54DA-414F-B90E-BE3970D3B476

**Fig 7:**
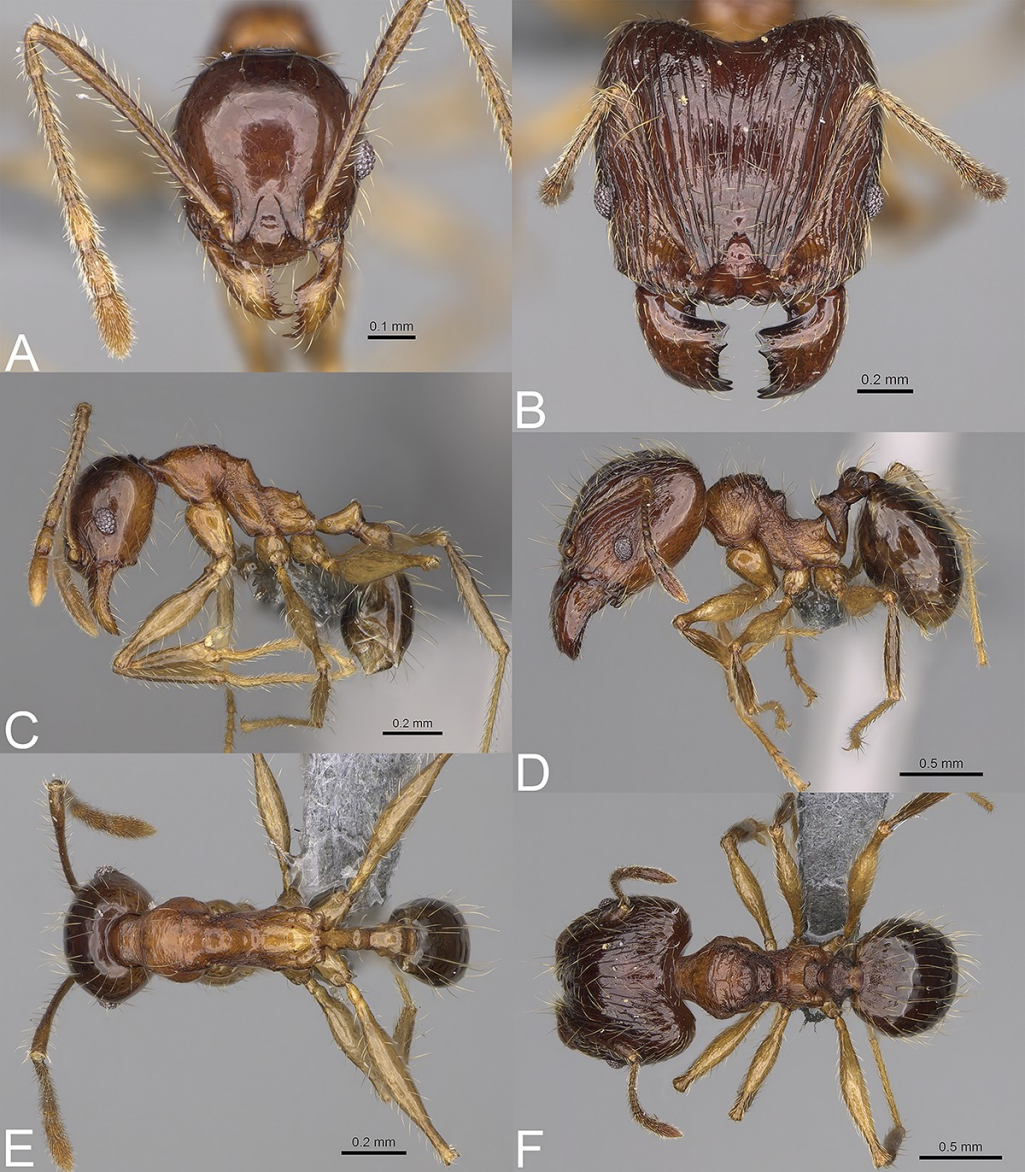
*Pheidole arivo*, full-face view (A), profile (C), and dorsal view (E) of paratype minor worker (CASENT0300266) and full-face view (B), profile (D), and dorsal view (F) of holotype major worker (CASENT0923263).

Figs 2B, 3B, 4B, 4I, 5B, 5I and 7A–7F

HOLOTYPE: 1s., Madagascar, Toamasina, Corridor Forestier Analamay-Mantadia, Ambohibolakely, -18.77898 48.36375, 918 m, 25-Nov-2012, rainforest, ex rotten log, B. L. Fisher et al. leg., BLF29805, CASENT0923263 (CASC). PARATYPES: 1w., data the same as holotype, CASENT0300266 (CASC); 1w., the same locality as holotype, ex dead twig above ground, BLF29802, CASENT0300263 (PBZT); 1w., 1s., the same locality as holotype, BLF29813, CASENT0300267 (MHNG).

#### Other material

Madagascar. Antsiranana: 1w., Réserve Spéciale de l'Ankarana, 22.9 km 224° SW Anivorano Nord, -12.90889 49.10983, 80 m; 3w., Sakalava Beach, -12.26972 49.39167, 10 m, R. Harin'Hala leg. Fianarantsoa: 3w., 43 km S Ambalavao, Rés. Andringitra, -22.23333 47, 825 m; 4w., 9.0 km NE Ivohibe, -22.42667 46.93833, 900 m; 1w., Fitovavy Fitovinany Region, District of Ifanadiana Belle vue area1200 m S of Ranomafana National Park entrance, -21.2665 47.42017, 1018 m; 1w., Forêt d'Ambalagoavy Nord, Ikongo, Ambatombe, -21.857068 47.37849, 625 m; 1w., Forêt de Vevembe, 66.6 km 293° Farafangana, -22.791 47.18183, 600 m. Toamasina: 4w., 6.3 km S Ambanizana, Andranobe, -15.6813 49.958, 25 m; 3w., 6.9 km NE Ambanizana, Ambohitsitondroina, -15.58506 50.00952, 825 m; 5w., 7 km SE Andasibe National Park Headquarters, -18.969856 48.465894, 1050 m; 1w., Ambanizana, Parc National Masoala, -15.57167 50.00611, 800–897 m, Andriamalala et. al. leg; 1w., Ambanizana, Parc National Masoala, -15.57167 50.00611, 900–950 m, Andriamalala et. al. leg; 2w., 1s., Ambatovy, 12.4 km NE Moramanga, -18.84963 48.2947, 1010 m; 3w., 2s., Ambatovy, 12.4 km NE Moramanga, -18.83937 48.30842, 1080 m; 1w., 1s., Ambatovy, 12.4 km NE Moramanga, -18.84773 48.29568, 1000 m; 1w., 1s., Ambatovy, 12.4 km NE Moramanga, -18.85813 48.28488, 1040 m; 1w., Analamay, -18.80623 48.33707, 1068 m; 4w., Andasibe National Park, botanic garden near entrance, West of ANGAP office, -18.925172 48.418651, 1025 m; 1w., Ankerana, -18.40062, 48.81311, 865 m; 2w., Ankerana, -18.40829 48.82107, 750 m; 1w., Ankerana, -18.4017 48.80605, 1035 m; 4w., F.C. Andriantantely, -18.695 48.81333, 530 m, H.J.Ratsirarson leg; 6w., F.C. Sandranantitra, -18.04833 49.09167, 450 m, H.J.Ratsirarson leg.; 1w., Forêt Ambatovy, 14.3 km 57° Moramanga, -18.85083 48.32, 1075 m; 1w., Manakambahiny Atsinanana, -17.75 48.71667, A. Pauly leg; 1w., Montagne d'Anjanaharibe, 18.0 km 21° NNE Ambinanitelo, -15.18833 49.615, 470 m; 1w., P.N. Mantadia, -18.79167 48.42667, 895 m, H.J.Ratsirarson leg.; 1w., Parc National de Zahamena, Onibe River, -17.75908 48.85468, 780 m; 3w., 1s., Reserve Betampona, Camp Rendrirendry 34.1 km 332° Toamasina, -17.924 49.19967, 390 m; 1w., Reserve Betampona, Camp Vohitsivalana, 37.1 km 338° Toamasina, -17.88667 49.2025, 520 m; 5w., Réserve Spéciale Ambatovaky, Sandrangato river, -16.77274 49.26551, 450 m; 1w., Réserve Spéciale Ambatovaky, Sandrangato river, -16.81739 49.29402, 360 m; 1w., 1s., Sahafina forest 11.4 km W Brickaville, -18.81445 48.96205, 140 m; 1w., Station forestière Analamazaotra, Analamazaotra 1.3km S Andasibe, -18.38466 48.41271, 980 m; 4w., 4s., Torotorofotsy, -18.87082 48.34737, 1070 m; 1w., Torotorofotsy, -18.87467 48.3725, 960 m, Woodhead & Vences leg. Toliara: 3w., 10 km NW Enakara, Rés. Andohahela, -24.56667 46.81667, 430 m; 1w., 13 km NW Enakara, Rés. Andohahela, -24.55 46.8, 1250 m.

#### Geographic range

Madagascar, Antsiranana and eastern verge of the island.

#### Diagnosis. Major worker

Head, in full-face view sub-rectangular, slightly widening posteriorly, with anterior and posterior margins convex. Margins of the head with dense, long and appressed to subdecumbent pilosity. Antennal scrobe sparsely costulate; interspaces between costulae smooth to indistinctly rugulate. Frons with thick and sparse costulae; interspaces between costulae mostly smooth or indistinctly rugulate. Sides posterolateral from eyes smooth. Occipital lobe with sparse and thick costulae; interspaces between costulae mostly smooth or indistinctly rugulate. Inner hypostomal tooth distinct, moderate, closely spaced, triangular, with the rounded top directed outward; outer hypostomal tooth lobe-like, distinctly lower than inner hypostomal teeth, top directed upward; median tooth absent. Mesosoma densely rugoreticulate; promesonotal dorsum with sparser sculpture but never smooth. Gaster with finely shagreened base of the first gastral tergite. Head and anterior mesosoma brown to reddish-brown; posterior mesosoma and legs yellowish brown; gaster brown. **Minor worker.** Head shiny; smooth or indistinctly microrugulate. Mesosoma sparsely punctate; promesonotum with weaker puncta and sometimes smooth dorsum; katepisternum mostly or entirely smooth. Head and gaster brown; mesosoma reddish to yellowish-brown; legs yellowish-brown.

#### Description. Major worker

Measurements (n = 9): HL: 1.3–1.47 (1.38); HW: 1.31–1.5 (1.39); SL: 0.75–0.86 (0.82); EL: 0.16–0.21 (0.18); WL: 1.14–1.26 (1.18); PSL: 0.18–0.2 (0.19); MTL: 0.78–0.86 (0.83); PNW: 0.51–0.58 (0.56); PTW: 0.19–0.25 (0.22); PPW: 0.48–0.57 (0.51); CI: 95.7–103.5 (99.4); SI: 54.2–65.3 (59.0); PSLI: 13.1–14.9 (13.9); PPI: 38.3–47.0 (42.7); PNI: 37.8–42.7 (40.2); MTI: 55.1–62.6 (59.9).

#### Head

In full-face view sub-rectangular, slightly widening posteriorly, with anterior and posterior margins convex (Fig 7B). In lateral view sub-oval. Inner hypostomal tooth visible. Margins of the head with dense, long and appressed to subdecumbent pilosity; head dorsum with dense, long and decumbent to erect pilosity. Antennal scrobe distinct but shallow; sparsely costulate; interspaces between costulae smooth to indistinctly rugulate. Frons with thick and sparse costulae; interspaces between costulae mostly smooth or indistinctly rugulate. Sides posterolateral from eyes smooth. Occipital lobe with sparse and thick costulae; interspaces between costulae mostly smooth or indistinctly rugulate. Gena with dense and thick costulae; interspaces between costulae smooth to indistinctly rugulate. Centre of clypeus smooth and shiny; lateral sides with indistinct rugulae; median notch present, wide, and deep; median longitudinal carina present; lateral longitudinal carinae absent. Scape, when laid back, exceeding the midlength of the head by two-fifths of its length; pilosity subdecumbent to erect (Fig 7B and 7D). Inner hypostomal tooth distinct, moderate and triangular, with the rounded top directed outward; outer hypostomal tooth lobe-like, distinctly lower than inner hypostomal teeth, top directed upward; inner and outer hypostomal teeth closely spaced and not connected by concavity; median tooth absent (Fig 2B). **Mesosoma.** In lateral view, mesonotal process distinct, tubercle-like; promesonotal groove absent; metanotal groove absent; propodeal spine moderate, with a wide base and acute top; humeral tubercle laterally weakly produced (Fig 7D). Surface shiny; densely rugoreticulate; promesonotal dorsum with sparser sculpture but never smooth. Pilosity dense, long, and erect (Fig 7D and 7F). **Petiole.** Shiny with fine and dense puncta; node smooth to finely punctate; in rear view dorsoventrally straight to slightly concave (Fig 7D and 7F). **Postpetiole.** Shiny and finely punctate; dorsum with reduced sculpture and sometimes smooth notch; in dorsal view oval; lateral margins medially with two short and dentate projections (Fig 7D and 7F). **Gaster.** Shiny; with finely shagreened base of first gastral tergite; pilosity dense, long, and erect (Fig 7D and 7F). **Colour.** Head and anterior mesosoma brown to reddish-brown; posterior mesosoma and legs yellowish brown; gaster brown (Fig 7D and 7F).

#### Description. Minor worker

Measurements (n = 10): HL: 0.57–0.73 (0.66); HW: 0.5–0.67 (0.59); SL: 0.71–0.82 (0.76); EL: 0.12–0.15 (0.13); WL: 0.76–0.98 (0.86); PSL: 0.04–0.11 (0.08); MTL: 0.57–0.7 (0.63); PNW: 0.34–0.44 (0.39); PTW: 0.08–0.12 (0.1); PPW: 0.15–0.21 (0.18); CI: 106.9–115.5 (111.4); SI: 117.6–142.5 (128.9); PSLI: 6.8–15.3 (11.6); PPI: 46.0–66.7 (55.9); PNI: 62.2–69.6 (65.4); MTI: 103.2–115.7 (107.8).

#### Head

Occipital margin slightly convex; occipital carina absent (Fig 7A). Pilosity sparse, long, and suberect to erect. Sculpture shiny and smooth; sometimes indistinctly microrugulate (Fig 7A and 7C). **Mesosoma.** In lateral view, promesonotum low and short, arched; promesonotal groove absent; metanotal groove indistinct; anterior mesonotum concave; propodeal spine small (Fig 7C). Sculpture shiny; sparsely punctate; promesonotum with weaker puncta and sometimes smooth dorsum; katepisternum mostly or entirely smooth. Pilosity sparse, long, and erect (Fig 7C and 7E). **Postpetiole.** Convex; in dorsal view distinctly widening posteriad (Fig 7C and 7E). **Gaster.** With sparse, erect pilosity (Fig 7C and 7E). **Colour.** Head and gaster brown; mesosoma reddish to yellowish-brown; legs yellowish-brown (Fig 7C and 7E).

#### Biology

The species was collected between 10–1250 m in elevation, in the rainforest, montane rainforest, tropical forest, dwarf littoral forest, montane forest, tropical dry forest and grassland. Nests were located in rotten logs, under stones and dead twigs above ground. Worker were collected from sifted litter.

#### Comments

*Pheidole arivo* is one of the most common members of the *fervens* group distributed in Antsiranana and the eastern part of the island. Its distribution overlaps with *P*. *mena*, *P*. *mamirapiratra*, and *P*. *indica*. Majors of *P*. *arivo* can be easily separated based on smooth sides posterolateral from eyes and distinct mesonotal process. Minors can be separated from *P*. *mena* based on the presence of puncta on mesosoma (minors of *P*. *mena* have mesosoma rugulate), and from *P*. *mamirapiratra*, and *P*. *indica* based on steep posterior mesonotum and entirely or mostly smooth katepisternum. In contrast, minors of *P*. *mamirapiratra* and *P*. *indica* have less steep posterior mesonotum and sculptured katepisternum.

#### Etymology

Malagasy for “thousand”. In reference to the wide distribution of the species.

### *Pheidole comosa* sp. nov.

urn:lsid:zoobank.org:act:77DB34F6-5A77-481C-9261-123F062A982F

**Fig 8:**
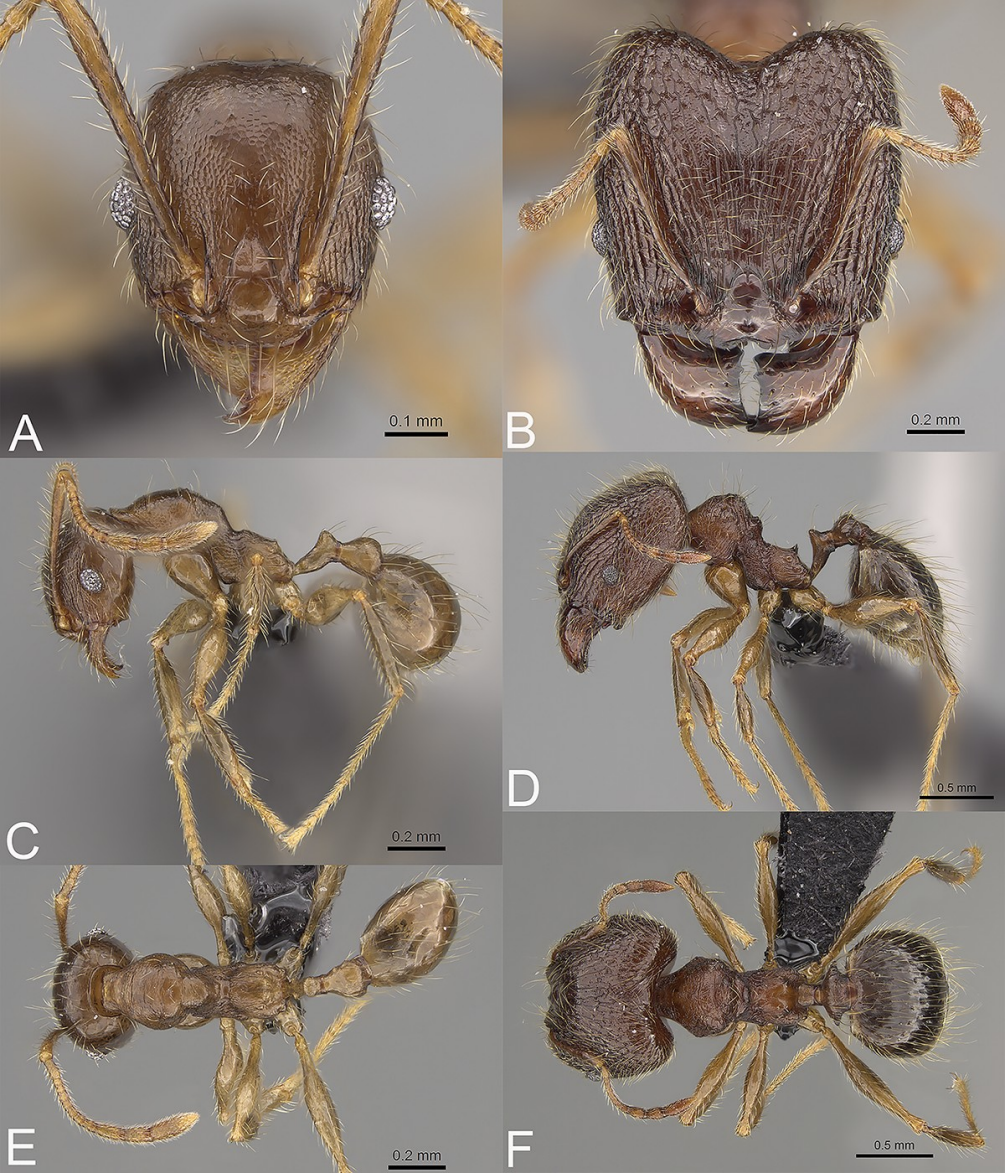
*Pheidole comosa*, full-face view (A), profile (C), and dorsal view (E) of paratype minor worker (CASENT0208811) and full-face view (B), profile (D), and dorsal view (F) of holotype major worker (CASENT0923278).

Figs 2C, 3C, 4C, 4J, 5C, 5J and 8A–8F

HOLOTYPE: 1s., Madagascar, Fianarantsoa, Anja Reserve, -21.85241 46.84579, 990 m, 14-Dec-2010, Degaded forest below granite out crop, under stone, B. L. Fisher et al. leg., BLF25953, CASENT0923278 (CASC). PARATYPES: 1w., the same data as holotype, CASENT0208811 (CASC); 2w., 1s., Anja Reserve, -21.85241 46.84579, 990 m, 14-Dec-2010, Degaded forest below granite out crop, under rootmat, litter on rock, B. L. Fisher et al. leg., BLF25938 (MHNG, PBZT).

#### Other material

Madagascar. Fianarantsoa: Toliara: 3 w., Forêt Classée d'Analavelona, 29.2 km 343° NNW Mahaboboka, -22.675 44.19, 1100 m; 1 w., Forêt Classée d'Analavelona, 33.2 km 344° NNW Mahaboboka, -22.64333 44.17167, 1300 m; 1 w., Parc National de Zombitse, 17.7 km 98° E Sakaraha, -22.88833 44.70167, 760 m.

#### Geographic range

Madagascar, Anja Reserve in Fianarantsoa, Forêt Classée d'Analavelona and Parc National de Zombitse in Toliara.

#### Diagnosis. Major worker

Head, in full-face view sub-rectangular, not widening posteriorly, with anterior and posterior margins convex. Margins of the head with dense, long and suberect to erect pilosity. Antennal scrobe costulate to rugocostulate; interspaces between costulae and rugocostulae distinctly rugopunctate. Frons with thick and dense costulae; interspaces between costulae indistinctly rugulate. Sides posterolateral from eyes and occipital lobe with dense and thick rugae; interspaces between rugae mostly smooth or indistinctly rugulate. Inner hypostomal tooth distinct, small, triangular, with rounded top directed inward; outer hypostomal tooth lobe-like, distinctly wider and higher than inner hypostomal teeth, top directed upward; inner and outer hypostomal teeth closely spaced and connected by concavity; median tooth absent. Mesosoma with sparse and thin rugulae; interspaces between rugulae smooth to indistinctly rugopunctate; promesonotal dorsum with sparser sculpture but never smooth. Gaster with a finely shagreened base of first gastral tergite. Body reddish-brown; legs yellowish-brown to brown. **Minor worker.** Head shiny and sparsely punctate; sides posterolateral from eyes entirely to mostly smooth. Mesosoma sparsely punctate; puncta sometimes weaker or absent on promesonotal dorsum, katepisternum and lateral sides of propodeum. Body brown; legs and antenna yellowish.

#### Description. Major worker

Measurements (n = 2): HL: 1.37, 1.64; HW: 1.34, 1.62; SL: 0.77, 0.82; EL: 0.16, 0.2; WL: 1.2, 1.44; PSL: 0.14, 0.21; MTL: 0.78, 0.87; PNW: 0.62, 0.76; PTW: 0.18, 0.18; PPW: 0.54, 0.7; CI: 102.4, 101.0; SI: 57.6, 50.7; PSLI: 10.4, 13.0; PPI: 33.2, 25.7; PNI: 46.3, 46.8; MTI: 57.9, 53.6.

#### Head

In full-face view sub-rectangular, not widening posteriorly, with anterior and posterior margins convex (Fig 8B). In lateral view sub-oval. Inner hypostomal tooth visible. Margins of the head with dense, long and suberect to erect pilosity; head dorsum with dense, long, decumbent to erect pilosity. Antennal scrobe distinct but shallow; costulate to rugocostulate; interspaces between costulae and rugocostulae distinctly rugopunctate. Frons with thick and dense costulae; interspaces between costulae indistinctly rugulate. Sides posterolateral from eyes and occipital lobe with thick rugae; interspaces between rugae mostly smooth or indistinctly rugulate. Gena with sparse and thick costulae; interspaces between costulae smooth to indistinctly rugulate. Centre of clypeus smooth and shiny, lateral margins with indistinct rugulae; median notch present, wide, and deep; median longitudinal carina present; lateral longitudinal carinae absent. Scape, when laid back, exceeding the midlength of head by two-fifths of its length; pilosity subdecumbent to erect (Fig 8B and 8D). Inner hypostomal tooth distinct, small, triangular, with rounded top directed inward; outer hypostomal tooth lobe-like, distinctly wider and higher than inner hypostomal teeth, top directed upward; inner and outer hypostomal teeth closely spaced and connected by concavity; median tooth absent (Fig 2C). **Mesosoma.** In lateral view, mesonotal process distinct, tubercle-like; promesonotal groove absent; metanotal groove absent; propodeal spine short, with a wide base and acute top; humeral tubercle laterally weakly produced (Fig 8D). Surface shiny with sparse and thin rugulae; interspaces between rugulae smooth to indistinctly rugopunctate; promesonotal dorsum with sparser sculpture but never smooth. Pilosity dense, long, and erect (Fig 8D and 8F). **Petiole.** Shiny with fine and dense puncta; node smooth to finely punctate; in rear view dorsoventrally straight to slightly concave; pilosity sparse and erect (Fig 8D and 8F). **Postpetiole.** Shiny and finely punctate; dorsum with reduced sculpture and sometimes smooth notch; in dorsal view oval, lateral margins medially with two dentate projections (Fig 8D and 8F). **Gaster.** Shiny, with a finely shagreened base of first gastral tergite; pilosity dense, long, and erect (Fig 8D and 8F). **Colour.** Reddish-brown; legs and yellowish-brown to brown (Fig 8D and 8F).

#### Description. Minor worker

Measurements (n = 8): HL: 0.55–0.64 (0.61); HW: 0.51–0.55 (0.53); SL: 0.64–0.71 (0.68); EL: 0.11–0.14 (0.12); WL: 0.75–0.81 (0.79); PSL: 0.05–0.1 (0.08); MTL: 0.5–0.54 (0.52); PNW: 0.34–0.36 (0.35); PTW: 0.09–0.11 (0.1); PPW: 0.2–0.24 (0.22); CI: 107.8–121.0 (114.1); SI: 117.1–134.0 (128.4); PSLI: 8.7–15.9 (13.4); PPI: 41.1–51.7 (45.8); PNI: 64.0–70.1 (66.3); MTI: 96.9–101.0 (98.8).

#### Head

Occipital margin slightly convex; occipital carina absent (Fig 8A). Pilosity sparse, long, and suberect to erect. Sculpture shiny and sparsely punctate; sides posterolateral from eyes entirely to mostly smooth (Fig 8A and 8C). **Mesosoma.** In lateral view, promesonotum low and short, slightly arched; promesonotal groove absent; metanotal groove distinct; anterior mesonotum slightly concave; propodeal spine indistinct to small (Fig 8C). Sculpture shiny; sparsely punctate; puncta sometimes weaker or absent on promesonotal dorsum, katepisternum and lateral sides of propodeum. Pilosity sparse, long, and erect (Fig 8C and 8E). **Postpetiole.** Convex; in dorsal view distinctly widening posteriad (Fig 8C and 8E). **Gaster.** With sparse, erect pilosity (Fig 8C and 8E). **Colour.** Brown; legs and antenna yellowish (Fig 8C and 8E).

#### Biology

The species was collected between 760–1300 m in elevation, in montane rainforest and tropical dry forest. Nest were located under stones, under rootmat, and in rotten log. Worker were collected from sifted litter.

#### Comments

*Pheidole comosa* is the most distinct member of the *fervens* group. The species is parapatric with *P*. *arivo* and differ from it, and all remaining members of the group, by majors with dense, long and suberect to erect pilosity on the margins of head, and costulate to rugocostulate antennal scrobe with distinctly rugopunctate interspaces. Its minors are the only ones within the group with distinctly punctate head.

#### Etymology

Latin for having long or abundant hair in reference to dense and long setosity of major workers.

### *Pheidole indica* Mayr, 1879

**Fig 9:**
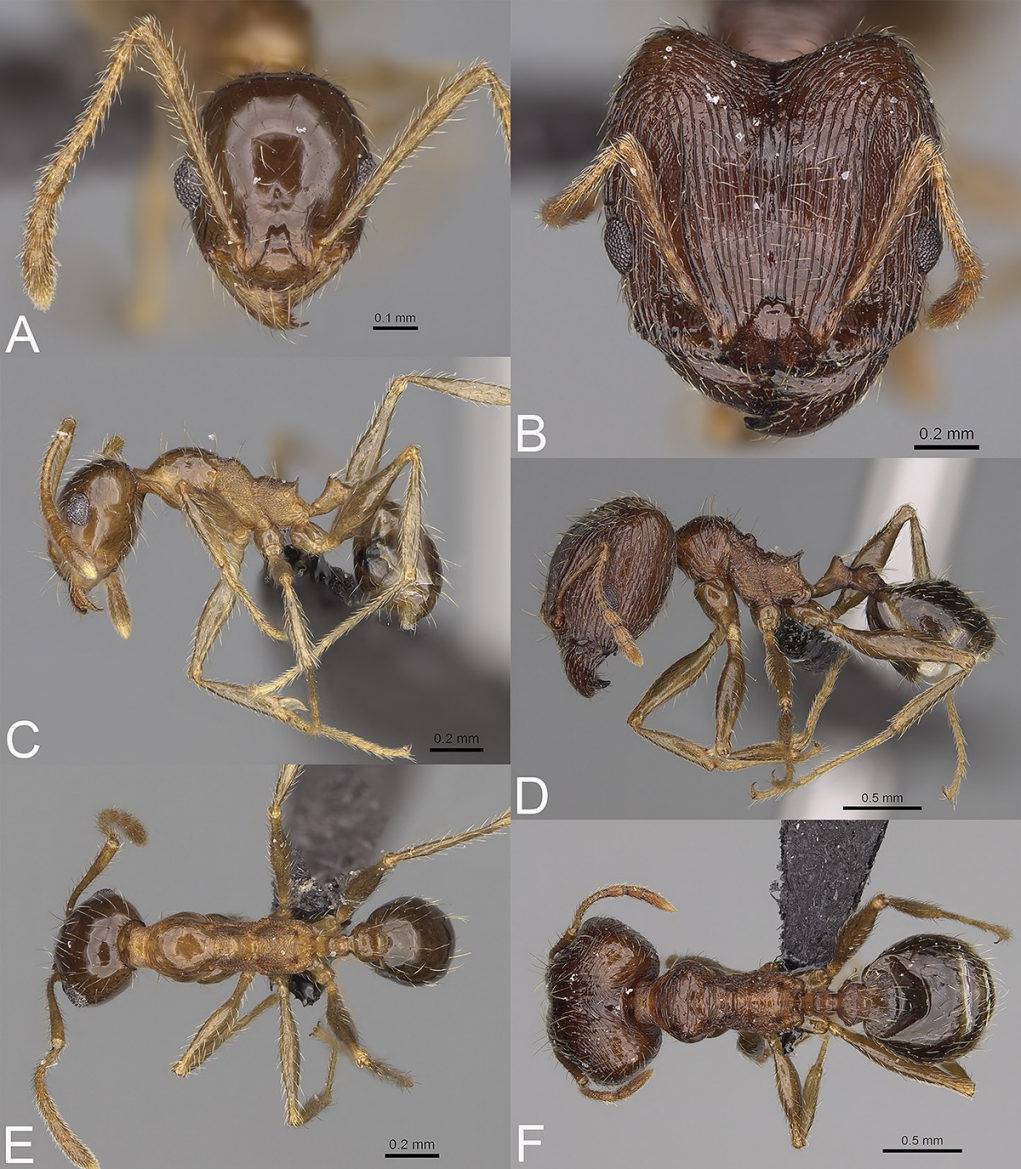
*Pheidole indica*, full-face view (A), profile (C), and dorsal view (E) of minor worker (CASENT0923269) and full-face view (B), profile (D), and dorsal view (F) of major worker (CASENT0122772).

Figs 2D, 3D, 4E, 4L, 5E, 5L and 9A–9F

*Pheidole indica* Mayr, 1879: 679 (s.w.q.) [20]

#### Other material

Madagascar. Antananarivo: 2w., 3s., Ankazobe, -18.31617 47.11583, 1241 m. 5w., 2s., 1q., Ambondromamy, -16.4375 47.1575, 64 m; 7w., 5s., 1q., Maevatanana, -16.94817 46.82767, 56 m; 7w., 2s., Mampikony, -16.09323 47.64278, 49 m; 2w., 2s., Port Berger, -15.56833 47.61925, 61 m; 5w., 4s., Majunga, -15.71783 46.317, 5 m. Toamasina: 1w., Toamasina-Ville, -18.15517 49.4095, 10 m. Toliara: 5w., 4s., Amboasary, -25.03883 46.3835, 25 m; 1w., 1s., Morondava, -20.2965 44.2815, 7 m.

#### Geographic range

Cosmopolitan species, on Madagascar known from urban and anthropogenic sites.

#### Diagnosis

See Fischer & Fisher 2013: 346 (under *P*. *teneriffana* Forel, 1893), and Sarnat et al. 2015: 45.

#### Biology

The species was collected between 1–1676 m in elevation, in gardens, date farms, secondary vegetation, urban area, palm tree plantations. Nest were located in soil, under stones, rotten logs, and Cattaleya leaf. Worker were collected from the ground and sifted litter.

#### Comments

Madagascar is a terra typica of *Pheidole voeltzkowii* Forel, 1894 a name currently considered as a junior synonym of *P*. *indica*. Personal investigation of type specimens of this species confirms its taxonomic position.

So far, *P*. *indica* has been recorded only from urban and anthropogenic sites of the island and its distribution overlaps only with *P*. *arivo*. However, majors of *P*. *indica* can be easily separated from other Madagascan members of the *fervens* group based on the combination of following characters: distinctly oval head in lateral view, sculptured margins posterolateral from eyes, distinct mesonotal process, antennal scrobe with costulae and smooth to indistinctly punctate interspaces, lateral margins of the head with sparse and appressed to subdecumbent setae, and entirely smooth gaster. Minors differ from remaining species based on entirely smooth head and promesonotum, steep posterior mesonotum and punctate katepisternum.

### *Pheidole mamirapiratra* sp. nov.

urn:lsid:zoobank.org:act:399DC8BD-1B0E-41CE-8C5C-6335F273325E

Figs 2E, 3E, 4F, 4M, 5F, 5M 10A–10F

**Fig 10:**
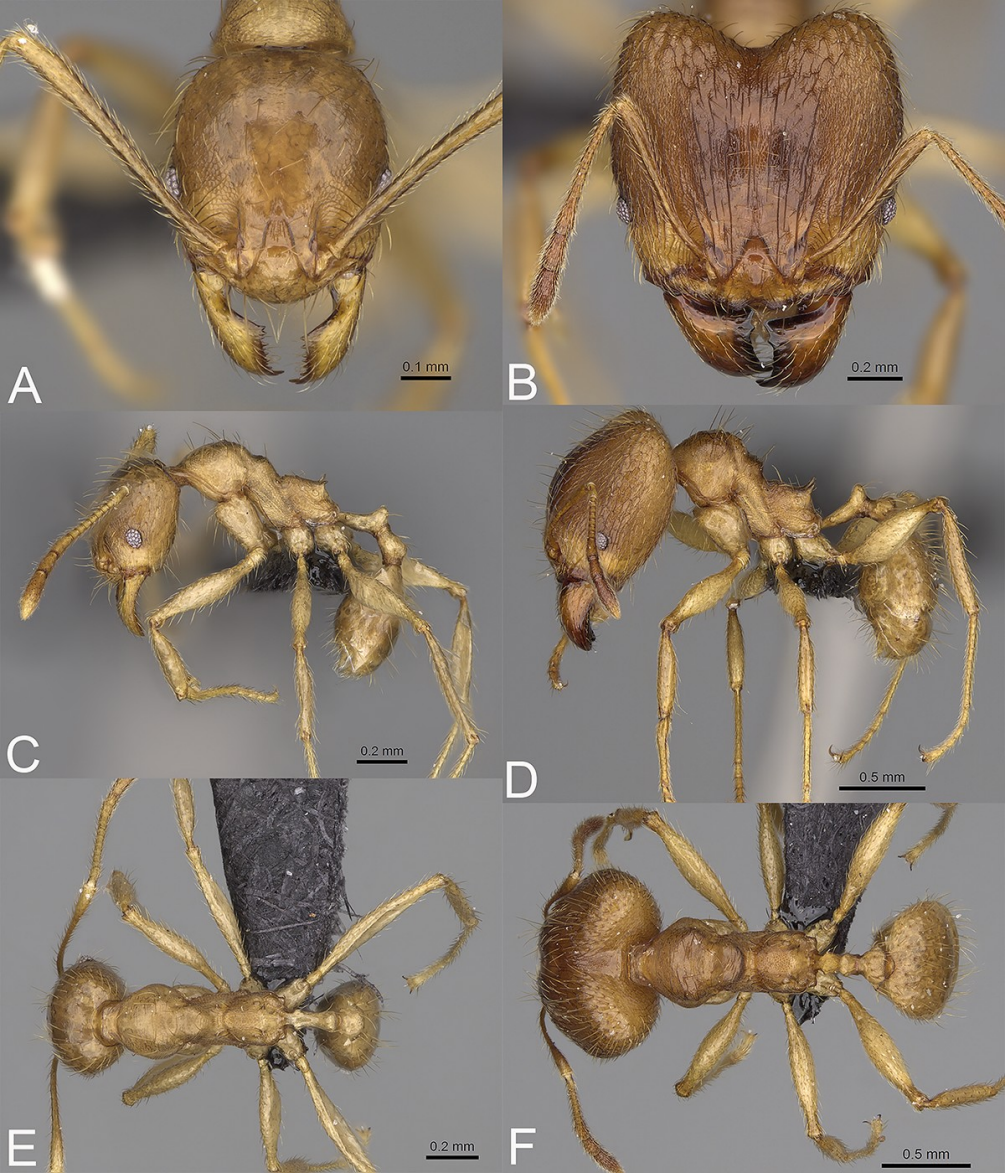
*Pheidole mamirapiratra*, full-face view (A), profile (C), and dorsal view (E) of paratype minor worker (CASENT0923268) and full-face view (B), profile (D), and dorsal view (F) of holotype major worker (CASENT0067219).

HOLOTYPE: 1s., Madagascar, Toamasina, Parc National Mananara-Nord, 7.1 km 261° Antanambe, -16.455 49.7875, 225 m, 15-Nov-2005, rainforest, ex rotten log, B. L. Fisher et al. leg., BLF12590, CASENT0067219 (CASC). PARATYPE: 1w., the same data as holotype, CASENT0923268 (CASC); 1w., 1s., the same locality as holotype., BLF12566, CASENT0066019 (PBZT); 1w., 1s., the same locality as holotype, BLF12578, CASENT0067502 (MHNG); 2w., 1s., 1m., the same locality as holotype, BLF12602, CASENT0067786, CASENT0067787 (CASC); 1w., 1s., the same locality as holotype, BLF12629, CASENT0067780 (CASC).

#### Other material

Madagascar. Antsiranana: 2w., 1s., 1m., Forêt Ambanitaza, 26.1 km 347° Antalaha, -14.67933 50.18367, 240 m; 1w., 1s., Parc National de Marojejy, Antranohofa, 26.6 km 31° NNE Andapa, 10.7 km 318° NW Manantenina, -14.44333 49.74333, 1325 m. Fianarantsoa: 4w., 3s., 1m., Réserve Speciale Manombo 24.5 km 228° Farafangana, -23.01583 47.719, 30 m. Toamasina: 1w., 1s., Analalava, 7.0 km 255° Mahavelona, -17.7095 49.454, 50 m; 17w., 17s., Montagne d'Akirindro 7.6 km 341° NNW Ambinanitelo, -15.28833 49.54833, 600 m; 12w., 3s., Montagne d'Anjanaharibe, 18.0 km 21° NNE Ambinanitelo, -15.18833 49.615, 470 m; 3w., 2s., 1q., Parc National de Zahamena, Besaky River, -17.75244 48.85321, 760 m; 2w., 2s., Parc National de Zahamena, Onibe River, -17.75908 48.85468, 780 m; 1w., Parc National de Zahamena, Sahavorondrano River, -17.75257 48.85725, 765 m; 5w., 5s., Parc National Mananara-Nord, 7.1 km 261° Antanambe, -16.455 49.7875, 225 m; 1w., 1s., Res. Ambodiriana, 4.8 km 306°Manompana, along Manompana river, -16.67233 49.70117, 125 m; 9w., 7s., 1q., Reserve Betampona, Camp Rendrirendry 34.1 km 332° Toamasina, -17.924 49.1997, 390 m; 8w., 8s., Reserve Betampona, Camp Vohitsivalana, 37.1 km 338° Toamasina, -17.88667 49.2025, 520 m; 6w., 5s., Réserve Nationale Intégrale Betampona, Betampona 35.1 km NW Toamasina, -17.91801 49.20074, 500 m; 3w., 3s., Réserve Spéciale Ambatovaky, Sandrangato river, -16.77274 49.26551, 450 m; 1w., 1m., Réserve Spéciale Ambatovaky, Sandrangato river, -16.76912 49.26704, 475 m; 1w., 1s., Réserve Spéciale Ambatovaky, Sandrangato river, -16.77468 49.26551, 355 m; 3w., 1m., Réserve Spéciale Ambatovaky, Sandrangato river, -16.7633 49.26692, 520 m; 2w., 1s., Réserve Spéciale Ambatovaky, Sandrangato river, -16.7755 49.26427, 430 m; 1w., Réserve Spéciale Ambatovaky, Sandrangato river, -16.81739 49.29402, 360 m; 3w., 3s., 1q., Réserve Spéciale Ambatovaky, Sandrangato river, -16.81745 49.2925, 400 m.

#### Geographic range

Madagascar, Cap Masoala in Antsiranana and north-eastern part of Toamasina, with a single record from Réserve Speciale Manombo in Fianarantsoa.

#### Diagnosis. Major worker

Head, in full-face view sub-rectangular, slightly widening posteriorly. Margins of the head with dense, long and suberect to erect pilosity. Antennal scrobe with dense network of microrugulae and additional sparse and thick costulae. Frons with thick and sparse costulae, interspaces mostly smooth or with indistinct and dense microrugulae. Sides posterolateral from eyes and occipital lobe with dense network of microrugulae and additional sparse and thick rugae. Inner hypostomal tooth distinct, large, dentate, with the rounded top directed upward; outer hypostomal tooth lobe-like, approximately as high and wide as inner teeth; median tooth absent. Mesosoma densely microrugulate; promesonotal dorsum with additional thick and transverse rugae and weaker microrugulae; sometimes lateral sides of pronotum also with weaker microrugulae. Gaster with an indistinctly shagreened base of first gastral tergite. Body yellow to yellowish-brown; head and mesosoma sometimes slightly darker than other parts of the body. **Minor worker.** Head shiny; indistinctly punctate; sides posterolateral from eyes smooth to indistinctly punctate. Mesosoma punctate; promesonotal dorsum with weaker puncta and additional short and transverse rugae; lateral sides of pronotum sometimes partially smooth. Body yellow to yellowish-brown, head and mesosomal dorsum sometimes darker, yellowish-brown.

#### Description. Major worker

Measurements (n = 10): HL: 1.21–1.39 (1.29); HW: 1.22–1.37 (1.27); SL: 0.78–0.86 (0.81); EL: 0.13–0.16 (0.15); WL: 1.14–1.26 (1.2); PSL: 0.19–0.22 (0.21); MTL: 0.75–0.86 (0.8); PNW: 0.49–0.55 (0.52); PTW: 0.13–0.17 (0.15); PPW: 0.31–0.37 (0.34); CI: 98.9–103.2 (101.4); SI: 61.2–66.5 (63.5); PSLI: 15.2–17.7 (16.2); PPI: 40.7–48.3 (44.9); PNI: 39.2–42.0 (40.7); MTI: 59.6–64.9 (63.3).

#### Head

In full-face view sub-rectangular, slightly widening posteriorly, with anterior and posterior margins slightly convex (Fig 10B). In lateral view sub-oval. Inner hypostomal tooth visible. Margins of the head with dense, long, suberect to erect pilosity; head dorsum with dense, long and decumbent to erect pilosity. Antennal scrobe distinct but shallow; densely microrugulate with additional sparse and thick costulae. Frons with thick and sparse costulae; interspaces between costulae mostly smooth or indistinctly microrugulate. Sides posterolateral from eyes and occipital lobe densely microrugulate with additional sparse and thick rugae. Gena with sparse and thick costulae; interspaces between costulae distinctly microreticulate. Centre of clypeus smooth and shiny; lateral margins with indistinct microreticulae; median notch present, wide, and deep; median longitudinal carina present; lateral longitudinal carinae absent. Scape, when laid back, exceeding the midlength of the head by two-fifths of its length; pilosity subdecumbent to suberect (Fig 10B and 10D). Inner hypostomal tooth distinct, large, dentate, with rounded top directed upward; outer hypostomal tooth lobe-like, approximately as high and wide as inner teeth; inner and outer hypostomal teeth closely spaced and not connected by concavity; median tooth absent (Fig 2E). **Mesosoma.** In lateral view, mesonotal process moderate, tubercle-like; promesonotal groove absent; metanotal groove indistinct; propodeal spine large, with wide base and acute top; humeral tubercle laterally weakly produced (Fig 10D). Surface shiny; densely microrugulate; promesonotal dorsum with additional thick and transverse rugae and weaker microrugulae; sometimes lateral sides of pronotum with weaker microrugulae. Pilosity dense, long, and erect (Fig 10D and 10F). **Petiole.** Shiny and indistinctly shagreened; node, in rear view dorsoventrally convex (Fig 10D and 10F). **Postpetiole.** Shiny and shagreened; dorsum with reduced sculpture and sometimes with a smooth notch; in dorsal view oval, lateral margins medially with two small, dentate projections (Fig 10D and 10F). **Gaster.** Shiny and with an indistinctly shagreened base of first gastral tergite; pilosity dense, long, and erect (Fig 10D and 10F). **Colour.** Yellow to yellowish-brown; head and mesosoma sometimes slightly darker than other parts of the body (Fig 10D and 10F).

#### Description. Minor worker

Measurements (n = 10): HL: 0.6–0.65 (0.62); HW: 0.5–0.54 (0.52); SL: 0.69–0.76 (0.73); EL: 0.1–0.11 (0.1); WL: 0.78–0.85 (0.79); PSL: 0.09–0.11 (0.1); MTL: 0.54–0.61 (0.58); PNW: 0.32–0.39 (0.37); PTW: 0.08–0.11 (0.09); PPW: 0.14–0.18 (0.16); CI: 115.5–126.3 (120.0); SI: 135.5–146.1 (140.8); PSLI: 14.2–17.4 (15.6); PPI: 47.2–63.6 (57.1); PNI: 62.7–74.9 (71.0); MTI: 104.7–115.7 (111.3).

#### Head

Occipital margin slightly convex; occipital carina absent (Fig 10A). Pilosity dense, long, and decumbent to suberect. Sculpture shiny; indistinctly punctate; sides posterolateral from eyes smooth to indistinctly punctate (Fig 10A and 10C). **Mesosoma.** In lateral view, promesonotum low and short, arched; promesonotal groove absent; metanotal groove distinct; anterior mesonotum distinctly concave; propodeal spine moderate and with a wide base (Fig 10C). Sculpture punctate; promesonotal dorsum with weaker puncta and additional short, transverse rugae; lateral sides of pronotum sometimes partially smooth. Pilosity dense, long, and erect (Fig 10C and 10E). **Postpetiole.** Convex; in dorsal view widening posteriad (Fig 10C and 10E). **Gaster.** With sparse, erect pilosity (Fig 10C and 10E). **Colour.** Yellow to yellowish-brown, head and mesosomal dorsum sometimes darker, yellowish-brown (Fig 10C and 10E).

#### Biology

The species was collected between 30–1325 m in elevation, in the rainforest, littoral rainforest, and montane rainforest. Nests were located in rotten logs and sticks on the ground, in soil, in rotting tree stumps, in the petiole of Melastomataceae, and dead branch above ground.

#### Comments

*Pheidole mamirapiratra* is known from area spread between Cap Masoala in Antsiranana and Toamasina city, with a single additional record from Réserve Speciale Manombo in Fianarantsoa and is sympatric with *P*. *arivo* and *P*. *mena*. However, morphologically *P*. *mamirapiratra* is most reminiscent of the introduced *P*. *indica*. Majors of *P*. *mamirapiratra* can be separated from other members of the *fervens* group based on a dense network of microrugulae and additional sparse and thick costulae on antennal scrobe and sculptured margins posterolateral from eyes. Minors can be separated from *P*. *mena* based on punctate mesosoma, from *P*. *arivo* based on punctate katepisternum and indistinctly punctate head; from *P*. *indica* based on indistinctly punctate head and indistinctly sculptured promesonotum.

#### Etymology

Malagasy for bright, in reference to the body colouration.

### *Pheidole mena* sp. nov.

urn:lsid:zoobank.org:act:59FBC697-A026-47EC-9338-8402676F916C

**Fig 11:**
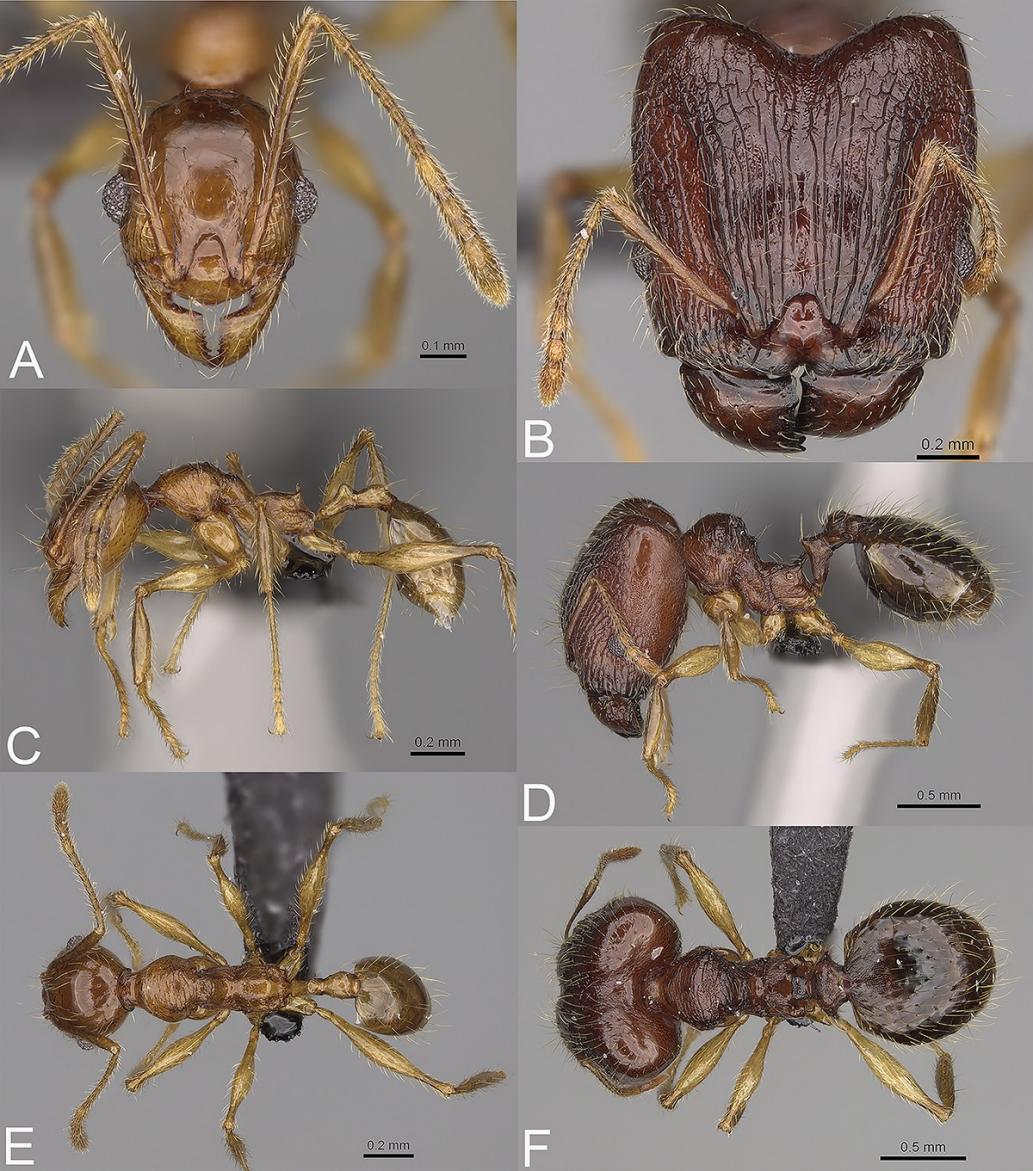
*Pheidole mena*, full-face view (A), profile (C), and dorsal view (E) of paratype minor worker (CASENT0235028) and full-face view (B), profile (D), and dorsal view (F) of holotype major worker (CASENT0134205).

Figs 2F, 3F, 4G, 4N, 5G, 5N and 11A–11F

HOLOTYPE: 1s., Madagascar, Antsiranana, Parc National Montagne d'Ambre, Roussettes, -12.52574 49.17238, 1025 m, 15-Nov-2007, montane rainforest, ex rotten log, B. L. Fisher et al. leg., BLF18303, CASENT0134205 (CASC). PARATYPE: 1w., the same data as holotype, CASENT0235028 (CASC).

#### Other material

Madagascar. Antsiranana: 6w., 6s., Antsiranana, Forêt de Binara, 9.1km 233° SW Daraina, -13.26333 49.60333; 2w., Makirovana forest, -14.16506 49.9477, 900 m; 1w., Makirovana forest, -14.104 50.03574, 225 m; 5w., 2s., Parc National de Marojejy, Manantenina River, 27.6 km 35° NE Andapa, 9.6 km 327° NNW Manantenina, -14.435 49.76, 775 m; 1s., Parc National de Marojejy, Manantenina River, 28.0 km 38° NE Andapa, 8.2 km 333° NNW Manantenina, -14.43667 49.775, 450 m; 1s., R.S. Manongarivo 17.3 km 218° SW Antanambao, -14.02167 48.41833, 1580 m; 1s., Réserve Spéciale de l'Ankarana, 22.9 km 224° SW Anivorano Nord, -12.90889 49.10983, 80 m. Toamasina: 2w., Montagne d'Anjanaharibe, 18.0 km 21° NNE Ambinanitelo, -15.18833 49.615, 470 m.

#### Geographic range

Madagascar, known from the northern part of Antsiranana.

#### Diagnosis. Major worker

Head, in full-face view sub-rectangular, not widening posteriorly to relatively straight. Margins of the head with dense, long and suberect to erect pilosity. Antennal scrobe densely costulate; interspaces between costulae smooth to distinctly punctate. Frons with thick and dense costulae; interspaces between costulae mostly smooth or indistinctly punctate. Sides posterolateral from eyes densely microrugulate; sculpture fading posteriad, and posteriormost parts usually smooth. Occipital lobe punctate with an additional network of sparse and thick rugae; sculpture weakening posteriorly. Inner hypostomal tooth distinct, large, dentate, with the rounded top directed indistinctly inward; outer hypostomal tooth lobe-like, approximately as high as inner teeth but distinctly wider; inner and outer hypostomal teeth closely spaced and connected by indistinct concavity; median tooth absent. Mesosoma with dense and thick rugoreticulae; interspaces between rugoreticulae smooth; promesonotal dorsum with sparser and more transverse sculpture. Gaster smooth; with a base of first gastral tergite shagreened. Head and mesosoma reddish-brown, gaster brown, legs and antenna yellow to yellowish-brown. **Minor worker.** Head shiny and smooth; interspaces between rugae surrounding antennal socket indistinctly rugulate. Mesosoma with thick and sparse rugae. Body yellow to yellowish-brown, head, gaster and mesosomal dorsum darker than the rest of the body.

#### Description. Major worker

Measurements (n = 8): HL: 1.47–1.76 (1.6); HW: 1.4–1.62 (1.52); SL: 0.76–0.83 (0.8); EL: 0.18–0.23 (0.2); WL: 1.13–1.3 (1.2); PSL: 0.21–0.28 (0.25); MTL: 0.75–0.89 (0.81); PNW: 0.59–0.67 (0.62); PTW: 0.19–0.26 (0.24); PPW: 0.54–0.73 (0.66); CI: 102.3–108.6 (105.6); SI: 51.5–54.7 (52.6); PSLI: 14.6–17.2 (15.5); PPI: 33.2–39.9 (37.0); PNI: 39.6–43.5 (41.0); MTI: 51.7–54.9 (53.0).

#### Head

In full-face view sub-rectangular, not widening posteriorly, with anterior and posterior margins slightly convex to relatively straight (Fig 11B). In lateral view sub-oval. Inner hypostomal tooth visible. Margins of the head with dense, long and suberect to erect pilosity; head dorsum with dense, long, decumbent to erect pilosity. Antennal scrobe distinct but shallow; densely costulate; interspaces between costulae smooth to distinctly punctate. Frons with thick and dense costulae; interspaces between costulae mostly smooth or indistinctly punctate. Sides posterolateral from eyes densely microrugulate; sculpture fading posteriad; posteriormost parts usually smooth. Occipital lobe punctate with an additional network of sparse and thick rugae; sculpture weakening posteriorly. Gena with dense and thick costulae; interspaces between costulae smooth to indistinctly punctate. Centre of clypeus smooth and shiny; lateral margins indistinctly microreticulate; median notch present, wide, and deep; median longitudinal carina present; lateral longitudinal carinae absent. Scape, when laid back, exceeding the midlength of the head by two-fifths of its length; pilosity subdecumbent to erect (Fig 11B and 11D). Inner hypostomal tooth distinct, large, dentate, with the rounded top directed indistinctly inward; outer hypostomal tooth lobe-like, approximately as high as inner teeth but distinctly wider; inner and outer hypostomal teeth closely spaced and connected by indistinct concavity; median tooth absent (Fig 2F). **Mesosoma.** In lateral view, promesonotum high; mesonotal process moderate, tubercle-like; promesonotal groove absent; metanotal groove absent; propodeal spine large, thin, with a wide base and acute top; humeral tubercle laterally weakly produced (Fig D). Surface shiny with dense and thick rugoreticulae; interspaces between rugoreticulae smooth; promesonotal dorsum with sparser and more transverse sculpture. Pilosity dense, long, and erect (Fig 11D and 11F). **Petiole.** Shiny and shagreened; node, in rear view dorsoventrally concave (Fig 11D and 11F). **Postpetiole.** Shiny and shagreened; dorsum with reduced sculpture and sometimes with smooth notch; in dorsal view oval, lateral margins medially with two distinct, dentate projections (Fig 11D and 11F). **Gaster.** Shiny and smooth; base of first gastral tergite shagreened; pilosity dense, long, and erect (Fig 11D and 11F). **Colour.** Head and mesosoma reddish-brown; gaster brown; legs and antenna yellow to yellowish-brown (Fig 11D and 11F).

#### Description. Minor worker

Measurements (n = 10): HL: 0.56–0.62 (0.59); HW: 0.48–0.52 (0.5); SL: 0.68–0.74 (0.7); EL: 0.13–0.14 (0.14); WL: 0.77–0.85 (0.81); PSL: 0.1–0.13 (0.1); MTL: 0.56–0.61 (0.58); PNW: 0.31–0.35 (0.34); PTW: 0.08–0.09 (0.08); PPW: 0.14–0.17 (0.16); CI: 110.2–122.4 (118.4); SI: 135.2–148.3 (140.7); PSLI: 15.0–20.2 (17.3); PPI: 50.0–59.9 (54.0); PNI: 61.3–72.3 (67.8); MTI: 112.9–121.2 (116.4).

#### Head

Occipital margin slightly convex; occipital carina absent (Fig 11A). Pilosity sparse, long, and decumbent to erect. Sculpture shiny and smooth; interspaces between rugae surrounding antennal socket indistinctly rugulate (Fig 11A and 11C). **Mesosoma.** In lateral view, promesonotum low and short, arched; promesonotal groove absent; metanotal groove distinct; anterior mesonotum slightly concave; posterior mesonotum smoothly declining towards propodeum; propodeal spine moderate and with wide base (Fig 11C). Sculpture with thick and sparse rugae. Pilosity sparse, long, and erect (Fig 11C and 11E). **Postpetiole.** Convex; in dorsal view slightly widening posteriad (Fig 11C and 11E). **Gaster.** With sparse, erect pilosity (Fig 11C and 11E). **Colour.** Yellow to yellowish-brown, head, gaster and mesosomal dorsum darker than the rest of the body (Fig 11C and 11E).

#### Biology

The species was collected between 80–1580 m in elevation, in tropical dry forest, montane rainforest and rainforest. Nest were located in rotten logs and rotten branch on the ground. Workers were collected from sifted litter.

#### Comments

*Pheidole mena* is distributed across the Antsiranana prefecture and is sympatric with *P*. *arivo* and *P*. *mamirapiratra*. Majors of *P*. *mena* can be easily separated based on reddish brown body coloration, presence of indistinct concavity connecting inner and outer hypostomal teeth, smooth posteriormost part of the margins of head, and more elongate and rectangular head shape in lateral view. Minors can be distinguished based on combination of the following characters: sparsely rugulate mesosoma, slightly concave anterior mesonotum, and smoothly declining towards propodeum posterior mesonotum.

#### Etymology

Malagasy for red, in reference to the body colouration of the major worker.

## Discussion

Madagascar hosts one of the highest numbers of endemic and threatened organisms on earth and is recognized as one of the top megadiversity regions [6, 21, 22]. The island has been isolated from continental Africa and Asia for more than 80 million years and sustains a wide range of ecoregions stretching from spiny deserts to tropical rainforest [6, 22]. *Pheidole* is listed among the five hyper-diverse and dominant ant genera of the island. Along with *Camponotus*, *Hypoponera*, *Strumigenys*, and *Tetramorium* contain more than 50% of the estimated ant species of the island [6]. Regardless of the abundance and diversity of those genera, their taxonomy had been in a deficient state until recent times. One can estimate that the understanding of the diversity of *Strumigenys* [23], *Camponotus* [24–27], and *Tetramorium* [28–32] is fairly complete.

Revision of Madagascan *Pheidole* was initiated in 2019, and the first species-group division of this genus was proposed by Salata & Fisher [3]. Authors of this publication reviewed 11 species-groups, redescribed 6 species, and described 46 taxa new to science. In the following publications, Salata & Fisher [4, 5] revised 62 additional species, representatives of the *sikorae* and *bessonii* species-groups. Results presented in this paper supplement the number of known and described *Pheidole* species by further 5 taxa. Thus, the total number of *Pheidole* known from the island is currently estimated at 124, and as many as 122 or 98% of them are known exclusively from Madagascar. While for the Malagasy region, the number increased to 135, and 131 or 97% of them are recorded only from this region. Our results confirm exceptional diversity and richness or Madagascan fauna [6, 22] and are in line with estimations provided by Fisher & Peeters [6], who stated that the endemism rate for ants known from Madagascar and surrounding islands reaches 98%. However, the number is still incomplete and is expected to increase together with forthcoming revisions of the two species-groups: *megacephala* and *lucida*.

Madagascar, as one of the top megadiversity regions, faces numerous threats. Among the most destructive are deforestation and forest fragmentation caused by agriculture, mining and logging [21, 33, 34], and introductions of invasive species [35]. Madagascan endemic species, alike other insular endemics, may have evolved in the absence of natural enemies and be more vulnerable to predation and competition [36]. Fisher & Peeters [6] noted that 41 out of 1281 ant species known from Madagascar are introduced. However, their impact on native fauna is still unstudied. *Pheidole indica*, so far, the only known introduced on Madagascar member of the *fervens* species-group, is not regarded as a major pest to agriculture or native ecosystems [8]. However, Sarnat et a. [8] predict that it could negatively impact native arthropods. On the island, *P*. *indica* is recorded only from urban and anthropogenic sites, and there is no record of its direct negative impact on native ant fauna. However, we predict that the species will be continuing to spread across the island following advancing degradation and deforestation of Madagascar. Herewith, it can indirectly negatively impact native species by occupying their niches in regions exposed to human activity.

## Supporting information

S1 Table Morphometric dataset. Morphometric characters of worker individuals of the *Pheidole fervens* species-group. Data are given in mm.
(XLSX)

S2 Table Distribution dataset. Distribution records of specimens of the *Pheidole fervens* species-group from Madagascar.
(XLSX)
